# MDM2 promotes CELF6 ubiquitination-dependent degradation to promote neuroblastoma cell proliferation

**DOI:** 10.1038/s41419-025-08048-3

**Published:** 2025-10-21

**Authors:** Zhenzhen Zhao, Bao Zhang, Xu Zhang, Changchun Li

**Affiliations:** 1 Department of surgical oncology Children’s Hospital of Chongqing Medical University, Chongqing, China; 2 National Clinical Research Center for Child Health and Disorders, Ministry of Education Key Laboratory of Child Development and Disorders, Chongqing, China; 3 Chongqing Key Laboratory of Structural Birth Defect and Reconstruction, Chongqing, China

**Keywords:** Paediatric cancer, Ubiquitylation

## Abstract

The ubiquitin–proteasome system plays a crucial role in neuroblastoma progression, yet the regulation of key degradation targets remains incompletely understood. By integrating transcriptomic and proteomic data, we identified nine candidate proteins, including CELF6, whose degradation is potentially mediated by ubiquitination. Survival analyses revealed that high CELF6 expression correlated with a favorable prognosis. Functional assays demonstrated that CELF6 suppresses neuroblastoma cell proliferation without affecting apoptosis. Mechanistically, the E3 ubiquitin ligase MDM2 directly interacts with CELF6, promoting its degradation via K48-linked ubiquitination. MDM2 overexpression accelerates CELF6 degradation, while its inhibition stabilizes CELF6 protein levels, an effect reversed by proteasome inhibitors. Furthermore, MDM2-driven neuroblastoma cell proliferation is dependent on CELF6 depletion. These findings establish MDM2 as a key regulator of CELF6 stability and highlight the MDM2–CELF6 axis as a potential therapeutic target in neuroblastoma.

## Introduction

Neuroblastoma is a highly heterogeneous pediatric malignancy in which dysregulation of protein homeostasis is increasingly recognized as a driver of tumor progression [1, 2]. The ubiquitin–proteasome system (UPS) plays a pivotal role in the controlled degradation of cellular proteins, and aberrations in this pathway have been implicated in various cancers [3, 4]. Despite advances in understanding neuroblastoma biology, the contribution of ubiquitination-dependent degradation to its pathogenesis remains poorly defined [2].

When the ubiquitin–proteasome degradation pathway of proteins is disturbed, substrate proteins will show changes in protein levels without changes in transcriptional levels [5, 6]. In other words, proteins that change in protein levels in tumor tissues but do not change in transcriptional levels are most likely caused by dysregulation of the ubiquitin–proteasome pathway during cancer development [7, 8]. Thanks to the development of transcriptomics and proteomics technologies, it has become possible to screen these potential dysregulated proteins in neuroblastoma [9–11]. This study identified CELF6 as a dysregulated protein caused by abnormalities in the ubiquitin proteasome pathway. CELF6 is an RNA-binding protein reported to be involved in RNA splicing, thereby regulating cell proliferation [12, 13]. However, there are fewer reports in cancer research, such as colorectal cancer [14], breast cancer [15], and lung cancer [16]. Therefore, exploring the abnormal ubiquitination degradation of CELF6 in neuroblastoma and the corresponding regulatory mechanism will help to gain a deeper understanding of the deep-level molecular mechanisms of neuroblastoma and the exploration of related targets.

In this study, we combined transcriptomic and proteomic analyses to identify proteins that are selectively degraded via ubiquitination in neuroblastoma. CELF6 stood out in subsequent clinical relevance analyses, with high CELF6 expression associated with improved patient outcomes, whereas its downregulation was associated with advanced disease stages. Cellular functional assays confirmed that CELF6 regulates cell proliferation without affecting apoptosis. Further investigation revealed that the E3 ubiquitin ligase MDM2 directly interacts with CELF6 and promotes its degradation via K48-linked ubiquitination, thereby driving cell proliferation. These results provide important insights into UPS-mediated regulation of neuroblastoma and identify the MDM2–CELF6 axis as a promising therapeutic target.

## Method

### Patients and human placenta collection

Tissue samples were collected from the Children’s Hospital Affiliated to Chongqing Medical University. The study was approved by the Institutional Review Board of Chongqing Medical University, and written informed consent was obtained from the parents or legal guardians, with patient assent when applicable. A total of ten samples were included, comprising five without MYCN amplification and five with MYCN amplification. All patients were treatment-naïve, and diagnoses were confirmed by biopsy. Samples were surgically resected, briefly rinsed with saline to remove blood, washed several times with phosphate-buffered saline (PBS), and then stored at −80 °C or fixed in 4% paraformaldehyde for subsequent analyses.

### Cell lines and reagents

HEK-293T, SK-N-SH (Non-MYCN amplified), SH-SY5Y (Non-MYCN amplified and cloned from SH-N-SH), IMR-32 (MYCN amplified) and SK-N-BE(2) (MYCN amplified) cell lines were obtained from ATCC (Manassas, VA, USA) and cultured in a humidified incubator at 37 °C in medium supplemented with 10% fetal bovine serum and antibiotics (penicillin, 100 U/mL; streptomycin, 100 U/mL).

### Molecular cloning and transfection

Human CELF6 (NM_001172684.2), MDM2 (NM_001145337.3), and ubiquitin (NM_001281716.1) cDNAs were PCR-amplified and subcloned into CMV, pTriEX, or pcDNA3.1 expression vectors. CELF6 expression was silenced using a human shRNA plasmid kit (Origene, Locus ID 60677). siRNA transfections were carried out using Lipofectamine 2000 (Invitrogen, USA), while plasmid transfections were performed with Lipofectamine 3000 (Invitrogen, USA) according to the manufacturers’ protocols. Detailed primer sequences and additional information are provided in Supplementary Tables 1–2.

### Real-time fluorescence quantitative PCR

Real-time fluorescence quantitative PCR was performed according to the previous study of the research group [17]. Total RNA was extracted using TRIzol reagent (Invitrogen). Two hundred nanograms of RNA were reverse transcribed with the Evo M-MLV RT Mix Kit (Bio-Rad, USA) and treated with gDNA Clean for qPCR (ACCURATE BIOLOGY, China), followed by quantification on a Bio-Rad CFX Connect system (Bio-Rad, USA). Target cDNAs were amplified using gene-specific primers and the SYBR Green Premix Pro Taq HS qPCR Kit II (ACCURATE BIOLOGY, China). Primer sequences, designed and synthesized by Shanghai Sangon Biotech Co., Ltd., are provided in Supplementary Table 3. Relative gene expression was calculated using the 2^^−ΔΔCT^ method, normalized to 18S rRNA.

### Cell proliferation assay

Cell proliferation was evaluated using CCK-8 and colony formation assays. For the CCK-8 assay, HT-29, HCT116, and Caco2 cells were seeded at 800 cells per well in 96-well plates. Optical density at 450 nm (OD450) was measured at 0, 12, 24, and 48 h post-seeding, following the manufacturer’s protocol. For colony formation, transfected cells were seeded at 500 cells per well in 6-well plates and cultured for 10–16 days. Colonies were fixed, stained with 0.2% crystal violet, imaged, and manually counted.

### Flow cytometry analysis

SK-N-SH and SH-SY5Y cells were harvested using EDTA-free trypsin upon reaching 80–90% confluence after experimental treatment. Apoptosis was quantified with the Annexin V-FITC apoptosis detection kit (KeyGEN BioTECH, Nanjing, China). For cell cycle analysis, cells were fixed in precooled 70% ethanol at –20 °C, then resuspended in 1 mL staining solution containing 20 μg/mL propidium iodide, 0.1% Triton X-100, and 0.2 mg/mL RNase A, and incubated at 37 °C for 15 min. Data acquisition and analysis were performed using standard protocols at the Central Laboratory of the Children’s Hospital Affiliated to Chongqing Medical University.

### Immunoblotting and immunoprecipitation

Immunoblotting was based on the study by Shao et al. [18]. For standard immunoblotting, proteins were extracted using 1% SDS lysis buffer. For immunoprecipitation, cells were lysed in buffer containing 50 mM Tris-HCl (pH 7.5), 0.1% SDS, 1% Triton X-100, 150 mM NaCl, 1 mM dithiothreitol, 0.5 mM EDTA, 100 mM PMSF, 100 μg/mL leupeptin, 1 mM aprotinin, 100 mM sodium orthovanadate, 100 mM sodium pyrophosphate, and 1 mM sodium fluoride. Lysates were clarified by centrifugation at 13,000 × *g*, and the supernatant (2 mg/mL protein) was incubated with magnetic beads conjugated to anti-Flag antibodies overnight at 4 °C. Immune complexes were washed three times with lysis buffer and analyzed by immunoblotting using the indicated antibodies (see Supplementary Table 4).

### Ubiquitination assay

HEK-293T cells were co-transfected with the specified plasmids (see figure legends). To inhibit deubiquitination, 10 μM MG-132 was added 6 h prior to protein collection. Cells were lysed in 6 M guanidine hydrochloride, and Flag-CELF6 was affinity-purified using Flag magnetic beads with incubation for 12 h at 4 °C. After three washes with lysis buffer, ubiquitination was detected by immunoblotting using anti-Flag (for Flag-CELF6) or anti-HA (for HA-tagged ubiquitin) antibodies. β-Actin served as a loading control.

### Immunohistochemistry

Neuroblastoma tissues were fixed in 4% paraformaldehyde overnight, dehydrated, embedded in paraffin, and sectioned at 4 μm. Sections were dewaxed, rehydrated, and subjected to antigen retrieval in 10 mM sodium citrate buffer (pH 6.0) at high temperature. Endogenous peroxidase activity was quenched with 3% hydrogen peroxide for 20 min, followed by blocking with 1% goat serum albumin for 20 min at room temperature. Sections were incubated with primary antibodies at 4 °C overnight, then with corresponding secondary antibodies for 30 min at room temperature. After washing with TBS, sections were stained with DAB and counterstained with hematoxylin. Images were captured using a light microscope.

### Acquisition of transcriptome and proteome data of neuroblastoma

Transcriptome data were obtained from the TARGET database (https://ocg.cancer.gov/programs/target; access date: October 25, 2024), comprising mRNA expression profiles from 159 samples, including 126 MYCN non-amplified and 33 MYCN-amplified cases. Proteome data were sourced from the R2 tool (https://hgserver1.amc.nl/cgi-bin/r2/main.cgi; access date: October 25, 2024) under the internal identifier “ps_avgpres_fw2010prot34_fw2010prot,” including 34 samples (12 MYCN-amplified and 22 non-amplified patients) [19].

### Screening of potential abnormal ubiquitination-modified proteins

Data were stratified by MYCN amplification status. Differential expression analysis was conducted using the “limma” package in R (version 4.2.3) [20]. Genes with an FDR < 0.05 and log₂ fold change >4 were classified as differentially expressed, whereas genes with |log₂ fold change| <0.05 were deemed non-differentially expressed. Differential proteins were defined by a fold change >1 and an FDR < 0.05. Results were visualized with volcano plots to display upregulated and downregulated genes relative to MYCN status. The online tool Venny (https://bioinfogp.cnb.csic.es/tools/venny/; accessed October 25, 2024) was used to identify overlaps between differentially expressed proteins and non-differentially expressed genes. Kaplan–Meier survival curves were generated in GraphPad Prism (version 9.5.1) to assess the prognostic significance of these proteins.

### Potential signaling pathway enrichment analysis

To elucidate the mechanisms underlying CELF6 in neuroblastoma metastasis, we performed gene ontology (GO) analysis—including biological process, cellular component, and molecular function—and Kyoto Encyclopedia of Genes and Genomes (KEGG) pathway enrichment analysis [21]. Background gene data were retrieved from the “org.Hs.eg.db” package in R. Differentially expressed genes (DEGs) were visualized and analyzed using the “clusterProfiler” package in R (version 4.1.3), with an FDR < 0.05 considered statistically significant [22].

### Data analysis and statistics

All experiments were conducted at least three times, and results are presented as Mean ± SD. Statistical analysis was performed using Prism software (GraphPad Software). P values were determined using two-tailed Student’s *t* tests, with significance indicated as follows: *P ≤ 0.05; **P ≤ 0.01; ***P ≤ 0.001; ****P ≤ 0.0001).

## Result

### Combining transcriptome and proteome to screen differentially expressed proteins depending on ubiquitination degradation pathway in neuroblastoma

**Fig. 1:**
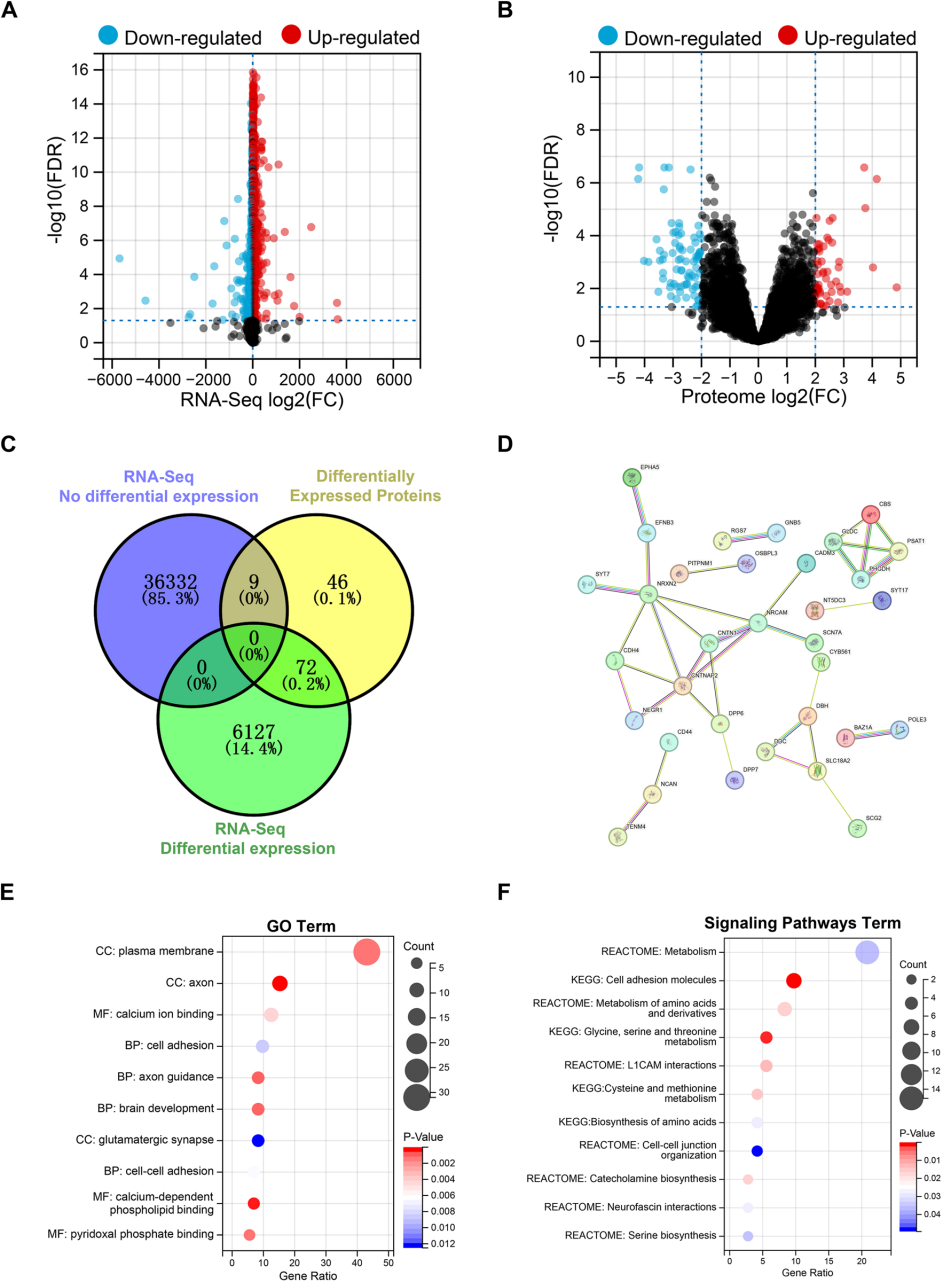
Screening differentially expressed proteins depending on ubiquitination degradation pathway in neuroblastoma. **A** Volcano plot of transcriptome differential analysis based on MYCN amplification status. **B** Volcano plot of proteome differential analysis based on MYCN amplification status. **C** Venn diagram to screen potential ubiquitination pathway degradation proteins. **D** Protein interaction network of 72 genes differentially expressed at both transcriptional and protein levels. **E** GO functional enrichment of 72 genes differentially expressed at both transcriptional and protein levels. The top 10 enriched items are arranged in descending order according to the number of gene enrichment (Counts). The color of the dots indicates the P value, and the size of the dots indicates the Counts. **F** KEGG and REACTOME pathway enrichment of 72 genes differentially expressed at both transcriptional and protein levels. The top 10 enriched items are arranged in descending order according to the number of gene enrichment (Counts). The color of the dots indicates the P value, and the size of the dots indicates the counts.

Proteins that rely on ubiquitination for degradation usually show changes in protein levels while RNA levels remain unchanged. Therefore, by combining RNA-seq and proteomic data, we obtained 6199 differential transcripts and 36,341 non-differential transcripts based on MYCN amplification status, and screened out 127 differential proteins (Fig. 1A, B). Further intersection results revealed nine potential ubiquitination-dependent degradation proteins, including PPBP, ITGB3, IGHV3OR16-12, BPI, CELF6, SDAD1, PKLR, EPB42, and LTF, and 72 genes that were differentially expressed at both transcript and protein levels (Fig. 1C). The subsequent protein interaction network showed that NRXN2, NRCAM, and CNTNAP2 seemed to be the hub proteins of the 72 genes (Fig. 1D). In addition, their cellular localization is mostly in plasma membrane and axon, and their molecular functions are related to calcium ion binding and pyridoxal phosphate binding, and are involved in biological processes such as cell adhesion, axon guidance, and brain development (Fig. 1E and Supplementary Table 5). In terms of signaling pathways, metabolism is the most important enriched pathway, followed by cell adhesion molecules, metabolism of amino acids and derivatives, and glycine, serine and threonine metabolism (Fig. 1F and Supplementary Table 5).

### High expression of CELF6 indicates better clinical prognosis

**Fig. 2:**
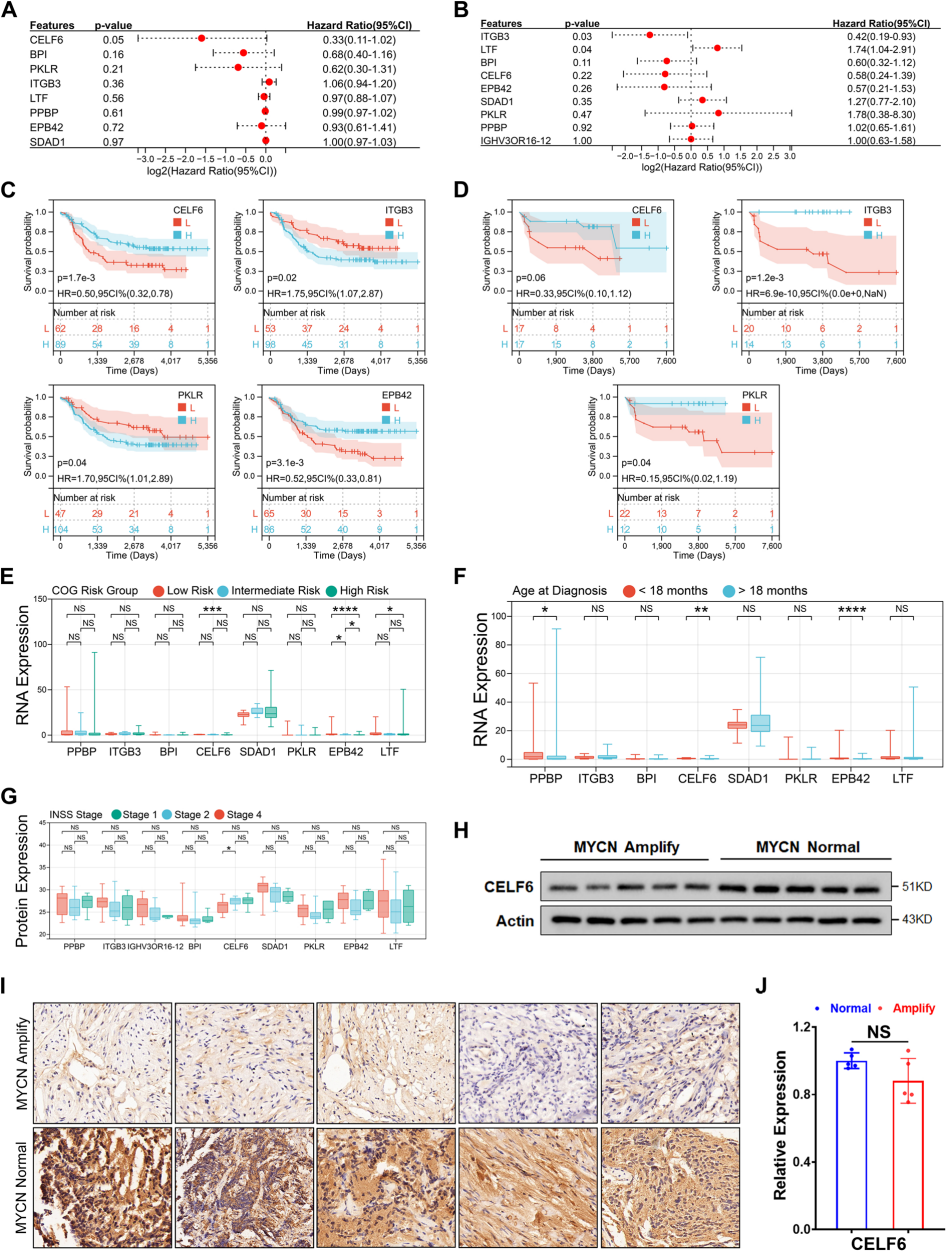
Correlation of CELF6 with prognosis in neuroblastoma. **A** Univariate Cox regression analysis based on gene transcription level. **B** Univariate Cox regression analysis based on protein expression level. **C** Kaplan–Meier analysis based on gene transcription level. **D** Kaplan–Meier analysis based on protein expression level. **E** Test for differences in gene transcription level based on COG risk stratification. The Wilcoxon signed-rank test was used to test the differences. NS: *p* > 0.05, *p < 0.05, ***p < 0.001, ****p < 0.0001. **F** Test for differences in protein expression level based on age at first diagnosis. The Wilcoxon signed-rank test was used to test the differences. The Wilcoxon signed-rank test was used to test the differences. NS: *p* > 0.05, *p < 0.05, **p < 0.01, ****p < 0.0001. **G** Test for differences in gene transcription level based on INSS stage. The Wilcoxon signed-rank test was used to test the differences. The Wilcoxon signed-rank test was used to test the differences.NS: *p* > 0.05, *p < 0.05. **H** Immunoblotting to detect the expression of CELF6 in clinical samples, including five MYCN amplified and five MYCN non-amplified samples. **I** Immunohistochemistry to detect the expression of CELF6 in clinical samples, including five MYCN amplified and five MYCN non-amplified samples. **J** Real-time fluorescence quantitative PCR to detect the expression of CELF6 in clinical samples, including five MYCN amplified and five MYCN non-amplified samples.

Univariate Cox regression analysis in both transcriptomic and proteomic cohorts identified CELF6, ITGB3, and LTF as candidate genes significantly associated with patient outcomes (Fig. 2A, B). Kaplan–Meier survival analysis further demonstrated that, within the transcriptomic cohort, CELF6, ITGB3, PKLR, and EPB42 effectively stratified patients by prognosis, whereas the remaining five genes did not (Figs. 2C and S1A). In the proteomic cohort, patients grouped by CELF6, ITGB3, and PKLR protein expression exhibited distinct survival outcomes, a pattern not observed for the other six proteins (Figs. 2D and S1B).

Moreover, differential expression of these nine candidate genes was observed when stratifying by clinical parameters. In transcriptomic data, CELF6 and EPB42 were expressed at lower levels in patients with higher COG risk, and individuals diagnosed after 18 years of age exhibited reduced expression of CELF6, EPB42, and PPBP (Fig. 2E, F). Regarding the MKI index, only BPI demonstrated differential expression, while CELF6 was the sole candidate showing significant variation with ploidy; notably, transcript levels of all candidate genes were not associated with recurrence (Fig. S1C–E). In the proteomic dataset, CELF6 protein levels were significantly reduced in patients with advanced INSS stages, whereas the other eight proteins did not show differences, and no correlation was found between initial diagnosis age and candidate protein levels (Figs. 2G and S1F).

Based on the robust association of both its transcript and protein levels with clinical outcomes, CELF6 was selected for further investigation. Ten neuroblastoma samples that did not receive chemotherapy were obtained from the clinic, including five MYCN amplified samples and five MYCN non-amplified samples, and the specific clinical information is shown in Table 1. Analysis of clinical samples confirmed that CELF6 protein expression was significantly lower in the MYCN-amplified group, as validated by immunoblotting and immunohistochemistry, while transcript levels remained comparable between groups (Fig. 2H–J).

**Table 1:** Sample information.

Sample ID	MYCN status	Gender	Age at diagnosis (Days)	INSS stage	MKI	Percent tumor (%)
NB01	Normal	Male	96	Stage 4	Low	60
NB02	Normal	Male	964	Stage 4	High	50
NB03	Normal	Female	538	Stage 3	Low	70
NB04	Normal	Male	287	Stage 2	Low	80
NB05	Normal	Male	67	Stage 1	Low	70
NB06	Amplified	Female	234	Stage 4	Low	90
NB07	Amplified	Female	939	Stage 3	Low	85
NB08	Amplified	Female	120	Stage 4	Low	90
NB09	Amplified	Male	342	Stage 4	Intermediate	80
NB10	Amplified	Male	432	Stage 3	High	90

### CELF6 inhibits neuroblastoma cell proliferation without affecting apoptosis

**Fig. 3:**
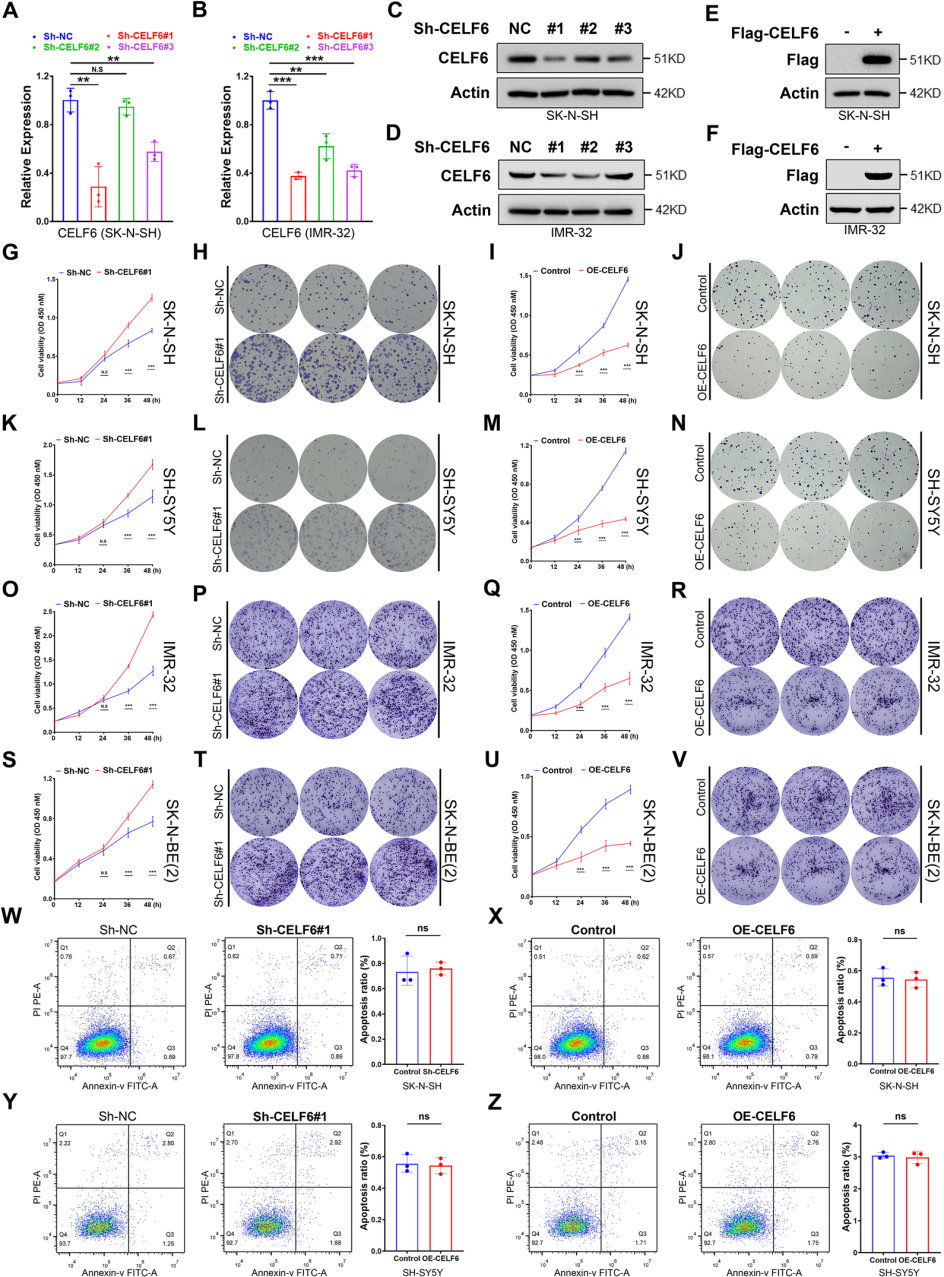
CELF6 regulates neuroblastoma cell function. **A**, **B** Real-time fluorescence quantitative PCR to detect the inhibitory efficiency of sh-CELF6 in SK-N-SH (**D**) and IMR-32 (**B**) cell line. Western blotting to detect the inhibitory efficiency of sh-CELF6 in SK-N-SH (**C**) and IMR-32 (**D**) cell line. Western blotting to detect whether Flag-CELF6 is successfully expressed in SK-N-SH (**E**) and IMR-32 (**F**) cell line. CCK-8 (**G**) or clone formation assay (**H**) to detect cell proliferation activity in SK-N-SH cells with or without stable inhibition of CELF6 expression. CCK-8 (**I**) or clone formation assay (**J**) to detect cell proliferation activity in SK-N-SH cells with or without overexpression of CELF6. CCK-8 (**K**) or clone formation assay (**L**) to detect cell proliferation activity in SH-SY5Y cells with or without stable inhibition of CELF6 expression. CCK-8 (**M**) or clone formation assay (**N**) to detect cell proliferation activity in SH-SY5Y cells with or without overexpression of CELF6 expression. CCK-8 (**O**) or clone formation assay (**P**) to detect cell proliferation activity in IMR-32 cells with or without stable inhibition of CELF6 expression. CCK-8 (**Q**) or clone formation assay (**R**) to detect cell proliferation activity in IMR-32 cells with or without overexpression of CELF6. CCK-8 (**S**) or clone formation assay (**T**) to detect cell proliferation activity in SK-N-BE(2) cells with or without stable inhibition of CELF6 expression. CCK-8 (**U**) or clone formation assay (**V**) to detect cell proliferation activity in SK-N-BE(2) cells with or without overexpression of CELF6 expression. **W** Flow cytometry to detect cell apoptosis ratio in SK-N-SH cells with or without stable inhibition of CELF6 expression. **X** Flow cytometry to detect cell apoptosis ratio in SK-N-SH cells with or without overexpression of CELF6 expression. **Y** Flow cytometry to detect cell apoptosis ratio in SH-SY5Y cells with or without stable inhibition of CELF6 expression. **Z** Flow cytometry to detect cell apoptosis ratio in SH-SY5Y cells with or without overexpression of CELF6 expression.

To elucidate the role of CELF6 in neuroblastoma cell function, we generated stable cell models with either CELF6 knockdown or overexpression (Figs. 3A–F and S3A–F). Effective CELF6 suppression was confirmed via immunoblotting and quantitative real-time PCR (Figs. 3A–D and S3A–D), whereas overexpression was validated by detecting the Flag tag in immunoblot analyses (Figs. 3E, F and S3E, F). Proliferation assays in SK-N-SH and SK-SY5Y cells revealed that CELF6 knockdown significantly enhanced cell proliferation, while its overexpression suppressed proliferation (Fig. 3G–N). Similarly, this was also true in IMR-32 and SK-N-BE(2) cells (Fig. 3O–V). In addition, modulation of CELF6 expression did not alter apoptosis rates in either cell line (Fig. 3W–Z).

### E3 ubiquitin ligase MDM2 reduces CELF6 protein levels via direct interaction

**Fig. 4:**
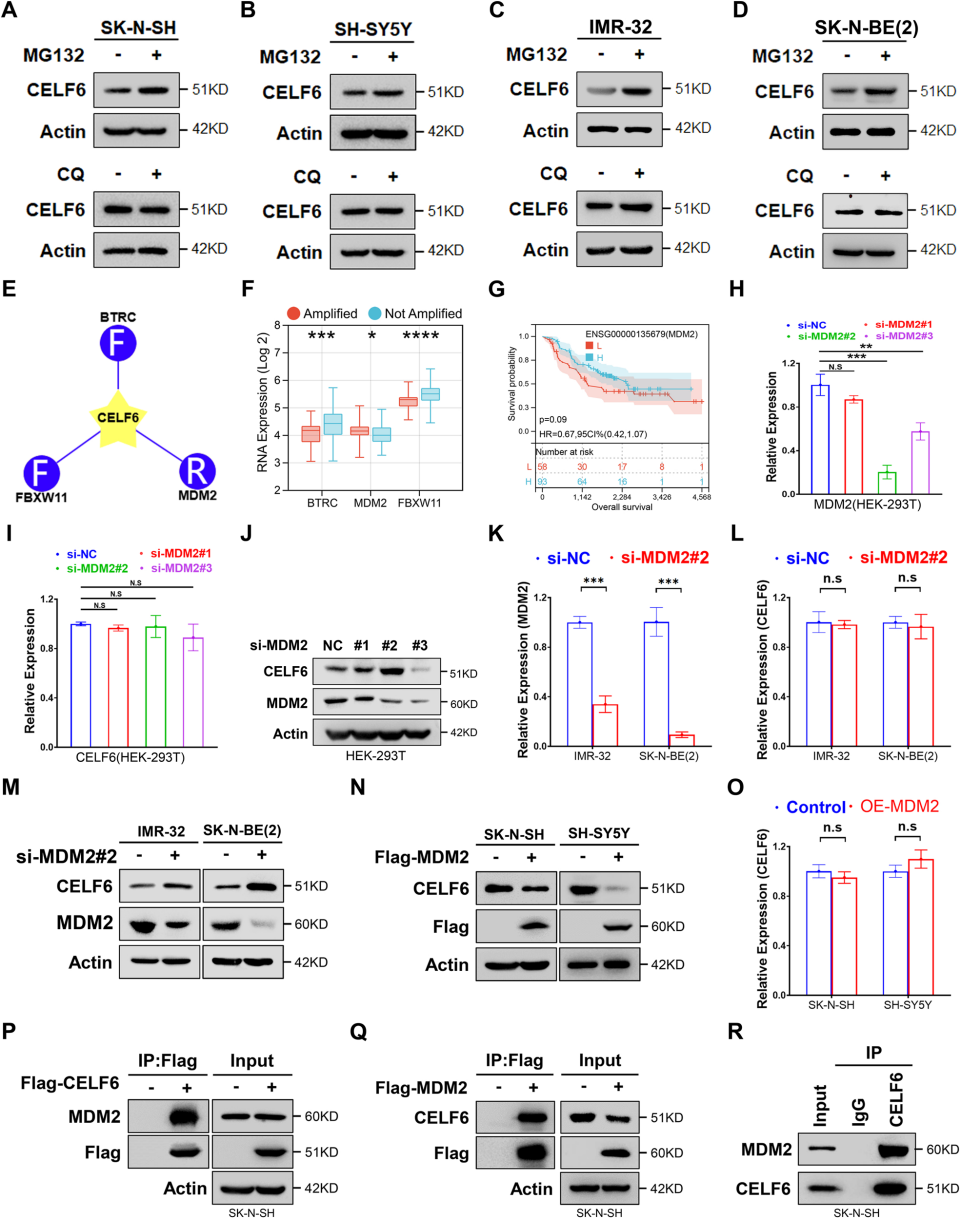
MDM2 is an E3 ubiquitin ligase for CELF6. **A** Immunoblotting to detect the expression of CELF6 in SK-N-SH with or without MG132 or CQ treatment. **B** Immunoblotting to detect the expression of CELF6 in SH-SY5Y with or without MG132 or CQ treatment. **C** Immunoblotting to detect the expression of CELF6 in IMR-32 with or without MG132 or CQ treatment. **D** Immunoblotting to detect the expression of CELF6 in SK-N-BE(2) with or without MG132 or CQ treatment. **E** Potential E3 ubiquitin ligases of CELF6 predicted by UbiBrowser. **F** Difference test of MDM2 transcription level, sample grouping based on MYCN hazard stratification. **G** Kaplan–Meier analysis based on RNA levels for MDM2. **H** Real-time fluorescence quantitative PCR to detect the transcriptional inhibition efficiency of si-MDM2 in HEK-293T. **I** Real-time fluorescence quantitative PCR to detect the effect of si-MDM2 on the transcriptional level of CELF6 in HEK-293T. **J** Immunoblotting to detect the effect of si-MDM2 on the protein level of CELF6. **K** Real-time fluorescence quantitative PCR to detect the transcriptional inhibition efficiency of si-MDM2#2 in IMR-32 and SK-N-BE(2). **L** Real-time fluorescence quantitative PCR to detect the effect of si-MDM2 on the transcriptional level of CELF6 in IMR-32 and SK-N-BE(2). **M** Immunoblotting to detect the protein level of CELF6 in IMR-32 and SK-N-BE(2) cells with or without si-MDM2#2. **N** Immunoblotting to detect the protein level of CELF6 in SK-N-SH and SH-SY5Y cells with or without MDM2 expression inhibition. **O** Real-time fluorescence quantitative PCR to detect the transcriptional level of CELF6 in SK-N-SH cells with or without MDM2 overexpression. **P** Co-immunoprecipitation to detect the interaction between Flag-CELF6 and endogenous MDM2. Cells were collected and lysed 48 h after transfection with Flag-CELF6, and immunoprecipitation was performed using 30 μl of Flag magnetic beads, followed by immunoblotting using the antibodies indicated in the figure. **Q** Co-immunoprecipitation to detect the interaction between Flag-MDM2 and endogenous CELF6. Cells were collected and lysed 48 h after transfection with Flag-CELF6, and immunoprecipitation was performed using 30 μl of Flag magnetic beads, followed by immunoblotting using the antibodies indicated in the figure. **R** Co-immunoprecipitation to detect the interaction between endogenous CELF6 and endogenous MDM2. The cells were collected and lysed 48 h after passage, and CELF6 antibody was added for immunoprecipitation according to the instructions. Protein A immunoaffinity gel beads were added 4 h later to pull down the CELF6 antibody, and then immunoblotting was performed using the antibodies shown in the figure.

To elucidate the degradation pathway of CELF6 in neuroblastoma cells, SK-N-SH, SH-SY5Y, IMR-32, and SK-N-BE(2) cells were treated with the proteasome inhibitor MG132 and the autophagy inhibitor chloroquine (CQ). MG132 treatment increased CELF6 protein levels in both cell lines, whereas CQ had no effect (Fig. 4A–D). Further analysis identified three candidate E3 ubiquitin ligases for CELF6. Further analysis identified three candidate CELF6 E3 ubiquitin ligases (Fig. 4E). Notably, BTRC and FBXW11 expression levels were lower in the MYCN amplified group (Fig. 4F), which was inconsistent with the lower CELF6 protein level in the MYCN amplified group (Fig. 2H), although survival analysis showed that BTRC and FBXW11, but not MDM2, were associated with neuroblastoma prognosis (Figs. 4G and S3A, B). In addition, MDM2 expression levels were lower in SK-N-SH and SH-SY5Y (MYCN non-amplified) and higher in IMR-32 and SK-N-BE(2) (MYCN amplified) (Fig. S3C), which is consistent with MDM2 being a direct transcriptional target of MYCN in neuroblastoma [23]. Silencing MDM2 with siRNA—confirmed by qRT-PCR (Fig. 4H, I)—did not alter CELF6 mRNA levels (Fig. 4J) but significantly elevated its protein levels (Fig. 4F), especially in the use of si-MDM2#2. CELF6 protein expression was increased (Fig. 4M). In contrast, overexpression of MDM2 in SK-N-SH and SH-SY5Y cells reduced CELF6 protein levels (Fig. 4N) but did not affect its transcription (Fig. 4O). In addition, exogenous Flag-tagged CELF6 co-immunoprecipitated with endogenous MDM2 (Figs. 4P and S3D), and conversely, Flag-tagged MDM2 co-immunoprecipitated with endogenous CELF6 (Fig. 4Q). Endogenous CELF6 and MDM2 also interacted, confirming a direct association between these proteins (Figs. 4R and S3E).

### MDM2 promotes CELF6 degradation via the ubiquitin-proteasome pathway

**Fig. 5:**
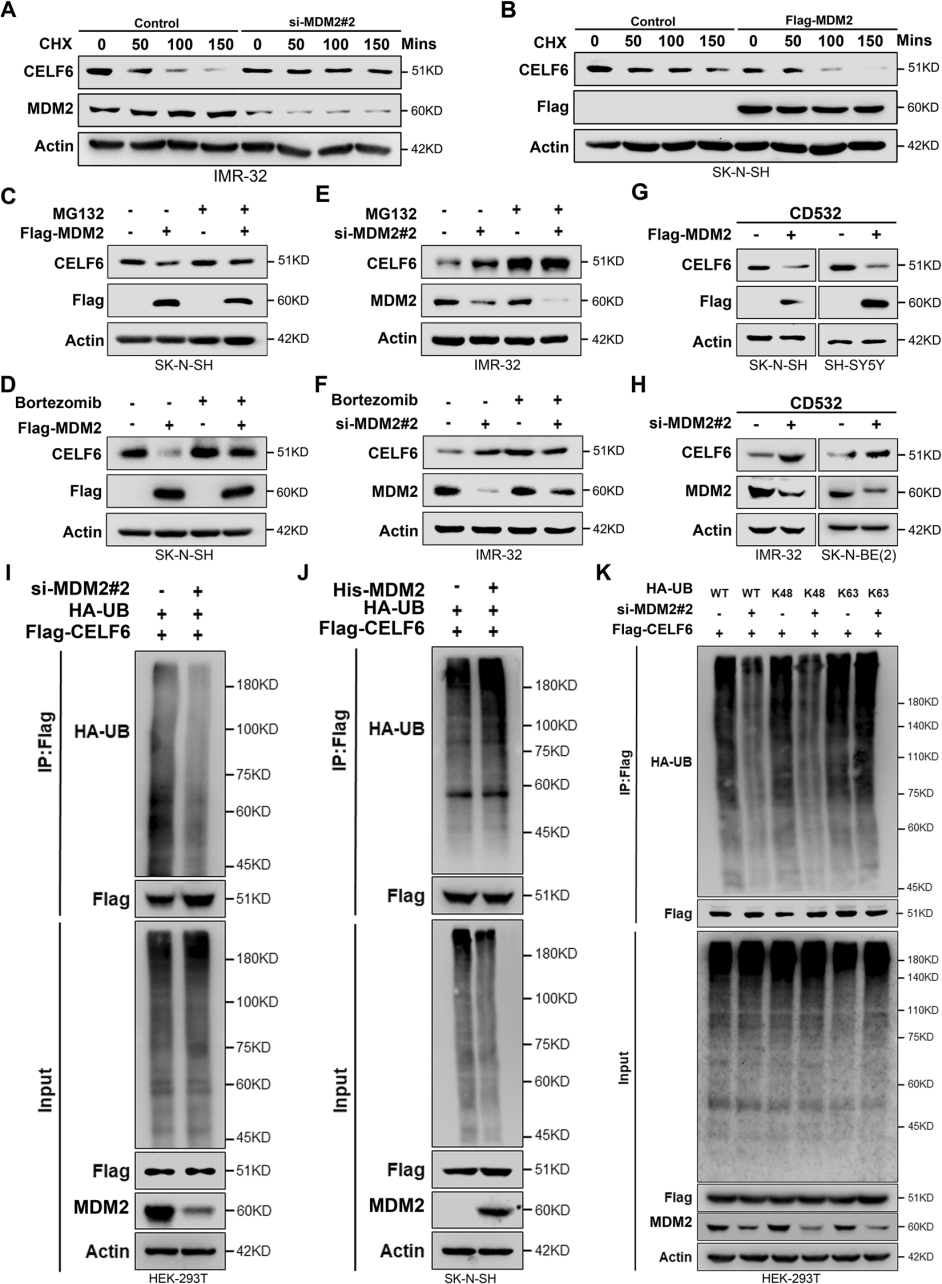
MDM2 promotes ubiquitination-dependent degradation of CELF6. **A** Western blot detection of CELF6 degradation rate in IMR-32 cells with or without overexpressed MDM2 expression, and CHX treatment time is indicated in the figure. **B** Western blot detection of CELF6 degradation rate in SK-N-SH cells with or without suppressed MDM2 expression, and CHX treatment time is indicated in the figure. Immunoblotting to examine the effect of overexpression of MDM2 on CELF6 protein levels in SK-N-SH cells with or without MG132 (**C**) or Bortezomib (**D**) treatment. **E**, **F** Immunoblotting to examine the effect of si-MDM2#2 on CELF6 protein levels in IMR-32 cells with or without MG132 (**C**) or Bortezomib (**D**) treatment. **G** Immunoblotting to examine the effect of Flag-MDM2 on CELF6 protein levels in SK-N-SH and SH-SY5Y cells with MYCN inhibitor (CD532). **H** Immunoblotting to examine the effect of si-MDM2#2 on CELF6 protein levels in IMR-32 and SK-N-BE(2) cells with MYCN inhibitor (CD532). **I**–**K** HEK-293T cells were transfected with the indicated plasmids for 48 h, treated with MG-132 (10 μM) for 6 h before cell lysate collection, immunoprecipitated with magnetic beads targeting Flag-tagged proteins, and then immunoblotted with the indicated antibodies. WT: wild-type ubiquitin molecule. K48: a mutant that only retains the 48th lysine of the ubiquitin molecule. K63: a mutant that only retains the 63rd lysine of the ubiquitin molecule.

To elucidate the role of MDM2 in regulating CELF6 stability, we employed cycloheximide (CHX) to inhibit protein synthesis and monitor CELF6 degradation. In contrast, in SK-N-SH, MDM2 overexpression significantly accelerated CELF6 protein degradation (Fig. 5B). Moreover, overexpression of MDM2 suppressed protein levels in SK-N-SH, which was abolished after treatment with MG132 or bortezomib (Fig. 5C, D). Similarly, the increase in CELF6 protein levels caused by reduced MDM2 levels in IMR-32 was also abolished by treatment with MG132 or bortezomib. Furthermore, a MYCN inhibitor (CD532) was used to exclude the possible differences caused by the different MYCN amplification status of the cell lines. It can be observed that the effect of MDM2 protein perturbation on CELF6 protein levels was not disrupted in both non-amplified and amplified neuroblastoma cell lines (Fig. 5G, H). Ubiquitination assays further revealed that MDM2 silencing significantly reduced CELF6 ubiquitination, whereas its overexpression enhanced this modification (Fig. 5I, J). Notably, siRNA-mediated inhibition of MDM2 decreased K48-linked ubiquitination of CELF6, without affecting K63-linked ubiquitination (Fig. 5K), consistent with the canonical role of K48-linked chains in directing proteins for proteasomal degradation [24].

### MDM2 promotes cell proliferation in a CELF6-dependent manner

**Fig. 6:**
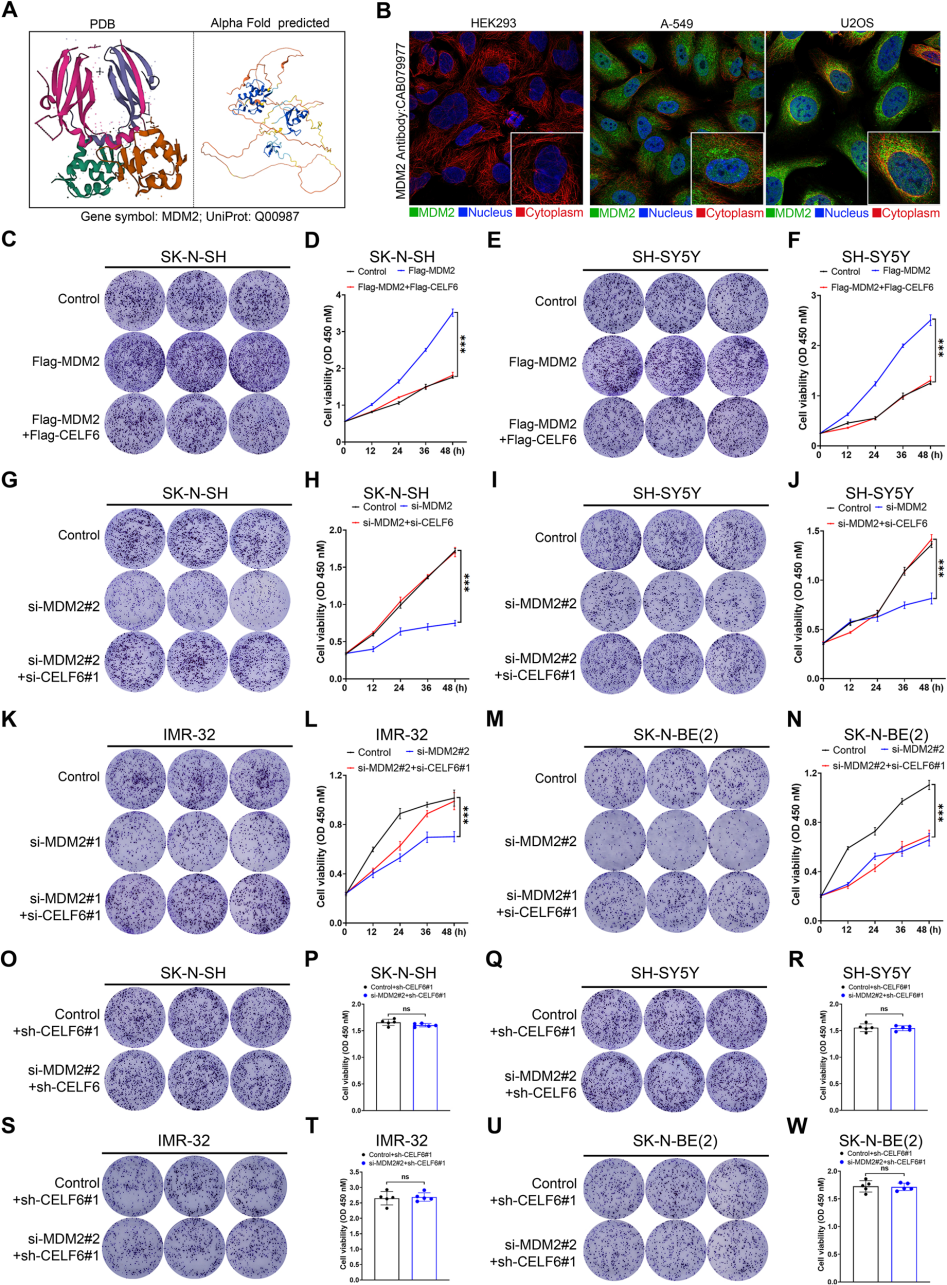
MDM2 depends on CELF6 to promote cell proliferation. **A** The spatial structure of MDM2 protein. The left one is from the PDB database, and the right one is predicted by Alphafold 3. **B** Cellular sublocalization of MDM2 in HEK293, A-549, and U2OS in the HPA database. The proliferation ability of SK-N-SH cells was detected by clone formation assay (**C**) and CCK-8 (**D**) 48 h after transfection of Flag-MDM2 or Flag-MDM2 and Flag-CELF6. The proliferation ability of SH-SY5Y cells was detected by clone formation assay (**E**) and CCK-8 (**F**) 48 h after transfection with Flag-MDM2 or Flag-MDM2 and Flag-CELF6. The proliferation ability of SK-N-SH cells was detected by clone formation assay (**G**) and CCK-8 (**H**) 48 h after transfection with si-MDM2#2 or si-MDM2#2 and si-CELF6#1. The proliferation ability of SH-SY5Y cells was detected by clone formation assay (**I**) and CCK-8 (**J**) 48 h after transfection with si-MDM2#2 or si-MDM2#2 and si-CELF6#1. The proliferation ability of IMR-32 cells was detected by clone formation assay (**K**) and CCK-8 (**L**) 48 h after transfection with si-MDM2#2 or si-MDM2#2 and si-CELF6#1. The proliferation ability of SK-N-BE(2) cells was detected by clone formation assay (**M**) and CCK-8 (**N**) 48 h after transfection with si-MDM2#2 or si-MDM2#2 and si-CELF6#1. The proliferation ability of SK-N-SH cells with stable inhibition of CELF6 expression was detected by clone formation assay (**O**) and CCK-8 (**P**) 48 h after transfection with or without si-MDM2#2#2. The proliferation capacity of SH-SY5Y cells stably inhibited by CELF6 expression was detected by clone formation assay (**Q**) and CCK-8 assay (**R**) 48 h after transfection or not with si-MDM2#2. The proliferation ability of IMR-32 cells with stable inhibition of CELF6 expression was detected by clone formation assay (**S**) and CCK-8 (**T**) 48 h after transfection with or without si-MDM2#2#2. The proliferation capacity of SK-N-BE(2) cells stably inhibited by CELF6 expression was detected by clone formation assay (**U**) and CCK-8 assay (**W**) 48 h after transfection or not with si-MDM2#2.

MDM2 is a 55.2 kDa E3 ubiquitin ligase (Fig. 6A), which was not associated with COG risk classification, INSS stage, or age at diagnosis (Fig. S3G, H). According to subcellular localization data from the Human Protein Atlas, MDM2 is expressed in both the nucleus and cytoplasm; however, it was not found in HEK293 cells (Fig. 6B), although MDM2 RNA and protein were found in HEK-293T cells (Fig. 4H, J). Cell proliferation assays revealed that MDM2 overexpression enhanced the proliferation of SK-N-SH and SH-SY5Y neuroblastoma cells, an effect that was abolished by Flag-CELF6 coexpression, as confirmed by both clonogenic assays and CCK-8 viability assays (Fig. 6C–F). In contrast, transient knockdown of CELF6 by siRNA, which was confirmed by real-time quantitative PCR and immunoblotting (Fig. S4A–E), reversed the cell proliferation inhibition caused by MDM2 knockdown (Fig. 6G–N). Notably, further inhibition of MDM2 did not produce additional antiproliferative effects in stably CELF6 knockdown neuroblastoma cells (Fig. 6O–W). Taken together, these findings suggest that MDM2 promotes neuroblastoma cell proliferation in a CELF6-dependent manner.

## Discussion

Our integrated multi-omics analysis reveals a critical regulatory axis in neuroblastoma in which ubiquitination-dependent degradation modulates cellular function independent of transcript levels. Notably, CELF6, a candidate tumor suppressor, exhibits reduced protein expression in MYCN-amplified tumors despite unchanged mRNA levels, underscoring the pivotal role of post-translational modifications in oncogenesis and implicating the ubiquitin–proteasome system in neuroblastoma pathogenesis.

MDM2, widely recognized for promoting tumorigenesis through p53 degradation [25], is a promising drug target with numerous inhibitors in development [26, 27]. In neuroblastoma, MDM2 is implicated in the clonal evolution of high-risk metastases [28], and the MDM2–p53 axis is critical to disease progression [29], presenting opportunities for novel therapeutic interventions [30, 31]. Although BTRC, FBXW11, and MDM2 were predicted as potential E3 ubiquitin ligases for CELF6, the expression trends of BTRC, FBXW11, and CELF6 in different MYCN amplification status groups were consistent, which did not conform to the hypothesis that BTRC or FBXW7 were E3 ubiquitin ligases for CELF6 (the E3 ubiquitin ligase promotes substrate degradation and the expression levels should be opposite). Therefore, MDM2 is the most potential E3 ubiquitin ligase for CELF6 in neuroblastoma cells, which was confirmed by subsequent experiments. MDM2 directly interacts with CELF6 and catalyzes its degradation via K48-linked ubiquitination. Perturbation of CELF6 expression mitigates the proliferative effects of MDM2, thereby expanding our understanding of MDM2’s function beyond p53 regulation. This may explain MDM2’s pervasive role in tumorigenesis, even in cancers with low TP53 mutation rates [32, 33].

CELF6 has been reported to inhibit cancer cell proliferation through intermediates such as p21 and FBP1 [12, 15], and our findings indicate that MDM2 disrupts this regulatory function. The accelerated turnover of CELF6 in the presence of MDM2 and its stabilization upon MDM2 knockdown highlight a finely tuned balance that governs cell proliferation. Restoration of CELF6, either by direct overexpression or MDM2 inhibition, significantly attenuates proliferation, suggesting that strategies to stabilize CELF6 may suppress tumor growth. Despite these advances, the molecular determinants dictating the specificity of MDM2-mediated CELF6 ubiquitination remain to be elucidated. Further studies should address whether additional cofactors or modifications modulate this interaction, and in vivo analyses are required to assess the therapeutic potential of targeting the MDM2–CELF6 axis.

Interestingly, MDM2 has been reported as a direct transcriptional target of MYCN in neuroblastoma [23], and MYCN amplification is a well-established determinant of patient prognosis, driving differences in cellular proliferation and differentiation [34]. In our study, consistent results were observed across four neuroblastoma cell lines—two with MYCN amplification and two without—demonstrating that MDM2-mediated ubiquitination of CELF6 and the CELF6-dependent regulation of cell proliferation occur regardless of MYCN status. Furthermore, we detected elevated MDM2 protein levels in MYCN-amplified neuroblastoma cell lines, consistent with its regulation as a direct MYCN target [23]. These findings suggest that the MYCN-driven transcriptional upregulation of MDM2, leading to enhanced ubiquitination and degradation of CELF6, may be one of the mechanisms underlying the increased proliferative capacity of MYCN-amplified neuroblastoma cells and their associated poor prognosis.

Overall, our study underscores the critical interplay between the ubiquitin–proteasome system and neuroblastoma progression. By defining the MDM2–CELF6 axis, we provide a compelling rationale for targeting this pathway as a therapeutic strategy, and our findings lay the groundwork for identifying additional ubiquitination-dependent regulators in neuroblastoma.

## Conclusion

In conclusion, our integrated transcriptomic and proteomic analyses reveal a pivotal role for ubiquitination in neuroblastoma. Notably, CELF6 acts as a tumor suppressor, with high expression correlating with improved clinical outcomes. We demonstrate that MDM2 directly interacts with and degrades CELF6 via K48-linked ubiquitination, thereby promoting cell proliferation. These findings define a novel MDM2–CELF6 axis in neuroblastoma pathogenesis, offering a promising therapeutic target for intervention.

## Supplementary information

Supplementary Information

Original Data

Original Data

## Data Availability

All data are available in the main text or the supplementary materials.
