# Supplementary material for: MDM2 promotes CELF6 ubiquitination-dependent degradation to promote neuroblastoma cell proliferation
Source: Cell Death Dis. 2025 Oct 21;16(1):736. doi: 10.1038/s41419-025-08048-3 (PMC12540984; doi:10.1038/s41419-025-08048-3)
Supplement: Supplementary file 1 — Supplementary Information [file 41419_2025_8048_MOESM1_ESM.docx]

**Supplementary information**

**MDM2 promotes CELF6 ubiquitination-dependent degradation to promote neuroblastoma cell proliferation**

**Zhenzhen Zhao^1,2,3^, Bao Zhang^1,2,3^, Xu Zhang^1,2,3^, and Changchun Li^1,2,3,*^**

1 Department of surgical oncology Children’s Hospital of Chongqing Medical University.

2 National Clinical Research Center for Child Health and Disorders, Ministry of Education Key Laboratory of Child Development and Disorders.

3 Chongqing Key Laboratory of Structural BirthDefect and Reconstruction.

* Correspondence: Changchun Li, Email: surgli@163.com.

Competing Interests: The authors have declared that no competing interest exists.

**Supplementary Figures**





**Figure S1. Correlation of CELF6 with prognosis in neuroblastoma.**

(A) Kaplan–Meier analysis based on gene transcription levels.

(B) Kaplan–Meier analysis based on protein expression levels.

(C) Test for differences in gene transcription levels based on MKI index groups. The Wilcoxon signed-rank test was used to test the differences. NS: *p*>0.05, **p* <0.05, ***p* <0.01.

(D) Test for differences in gene transcription levels based on whether the sample relapsed. The Wilcoxon signed-rank test was used to test the differences. NS: *p*>0.05.

(E) Test for differences in gene transcription levels based on polyploidy status. The Wilcoxon signed-rank test was used to test the differences. NS: *p*>0.05, **p* <0.05.

(F) Test for differences in gene protein expression levels based on age at first diagnosis. NS: *p*>0.05.


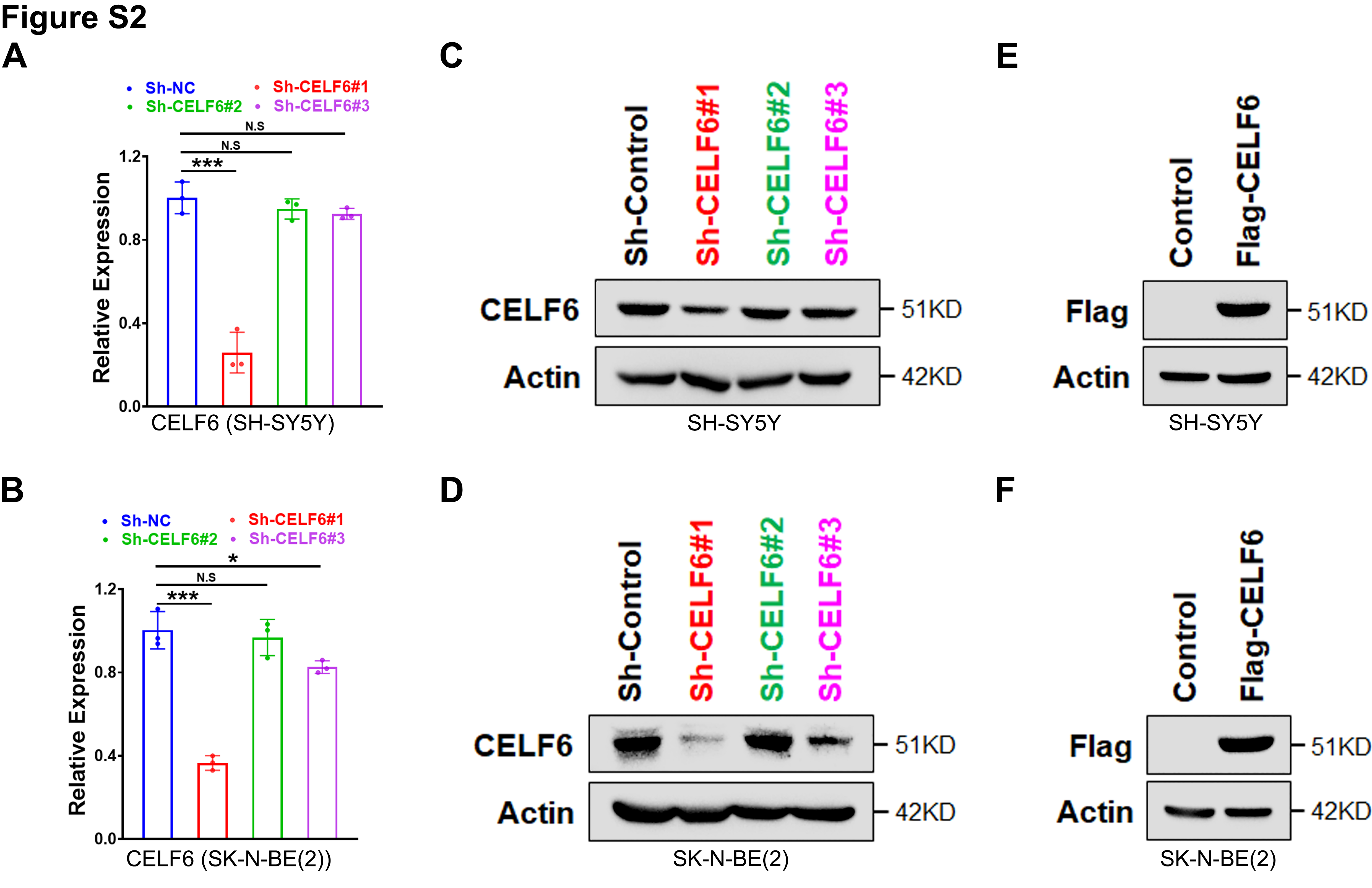


**Figure S2. Gene regulation efficiency of CELF6 in neuroblastoma cells.**

(A-B) Real-time fluorescence quantitative PCR to detect the inhibitory efficiency of sh-CELF6 in SH-SY5Y (A) and SK-N-BE(2) (B) cell line.

(C-D) Western blotting to detect the inhibitory efficiency of sh-CELF6 in SH-SY5Y (C) and SK-N-BE(2) (D) cell line.

(E-F) Western blotting to detect whether Flag-CELF6 is successfully expressed in SH-SY5Y (E) and SK-N-BE(2) (F) cell line.


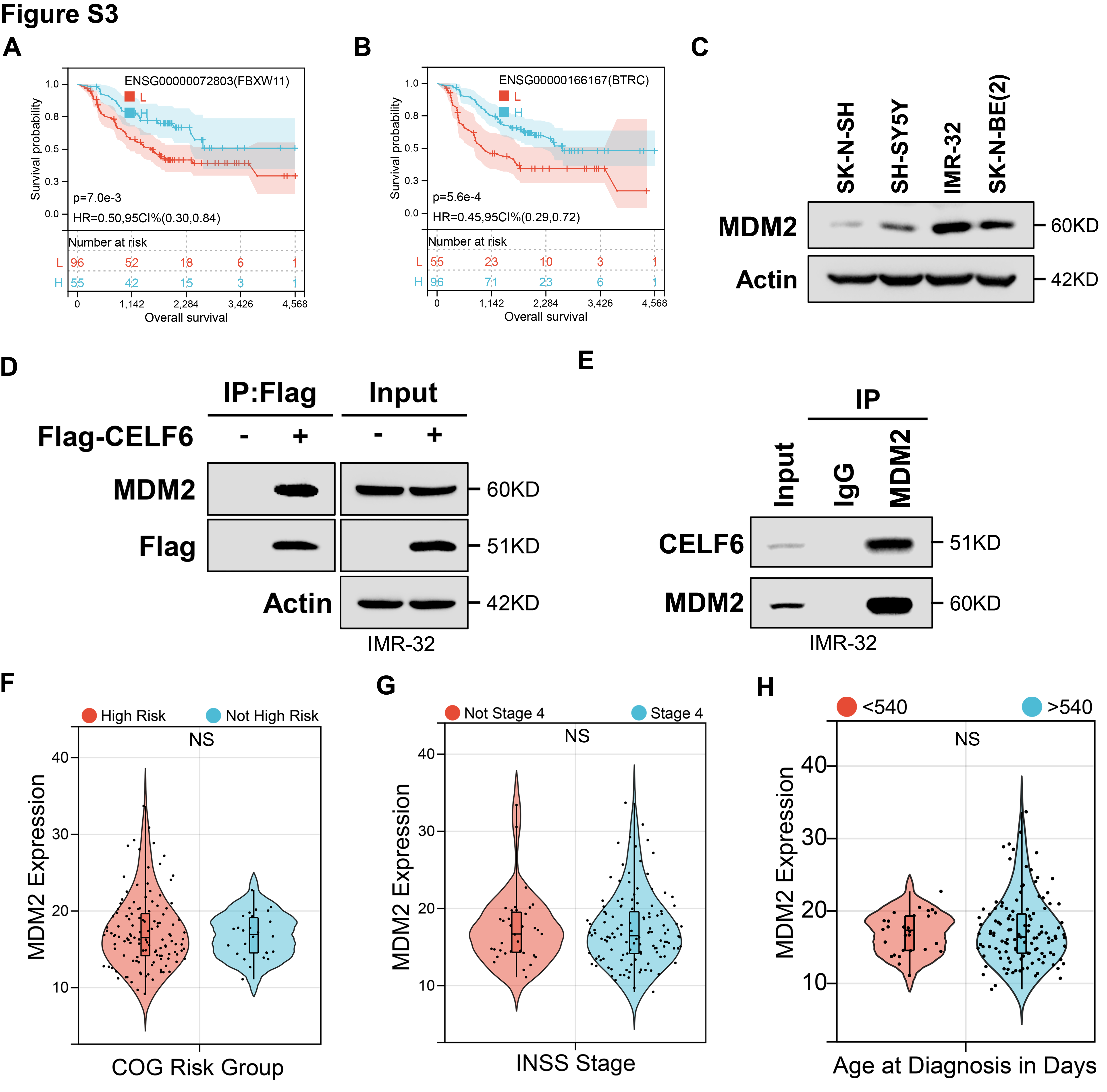


**Figure S3. MDM2 is an E3 ubiquitin ligase for CELF6.**

(A-B) Kaplan–Meier analysis based on RNA levels for FBXW11 (A) or BTRC (B).

(C) Immunoblotting to detect MDM2 protein levels in four neuroblastoma cell lines.

(D) Co-immunoprecipitation to detect the interaction between Flag-CELF6 and endogenous MDM2. Cells were collected and lysed 48 hours after transfection with Flag-CELF6, and immunoprecipitation was performed using 30ul of Flag magnetic beads, followed by immunoblotting using the antibodies indicated in the figure.

(E) Co-immunoprecipitation to detect the interaction between endogenous CELF6 and endogenous MDM2. The cells were collected and lysed 48 hours after passage, and MDM2 antibody was added for immunoprecipitation according to the instructions. Protein A immunoaffinity gel beads were added 4 hours later to pull down the MDM2 antibody, and then immunoblotting was performed using the antibodies shown in the figure.

(F) Difference test of MDM2 transcription level, sample grouping based on COG hazard stratification.

(G) Difference test of MDM2 transcription level, sample grouping based on INSS stage.

(H) Difference test of MDM2 transcription level, sample grouping based on age at first diagnosis.


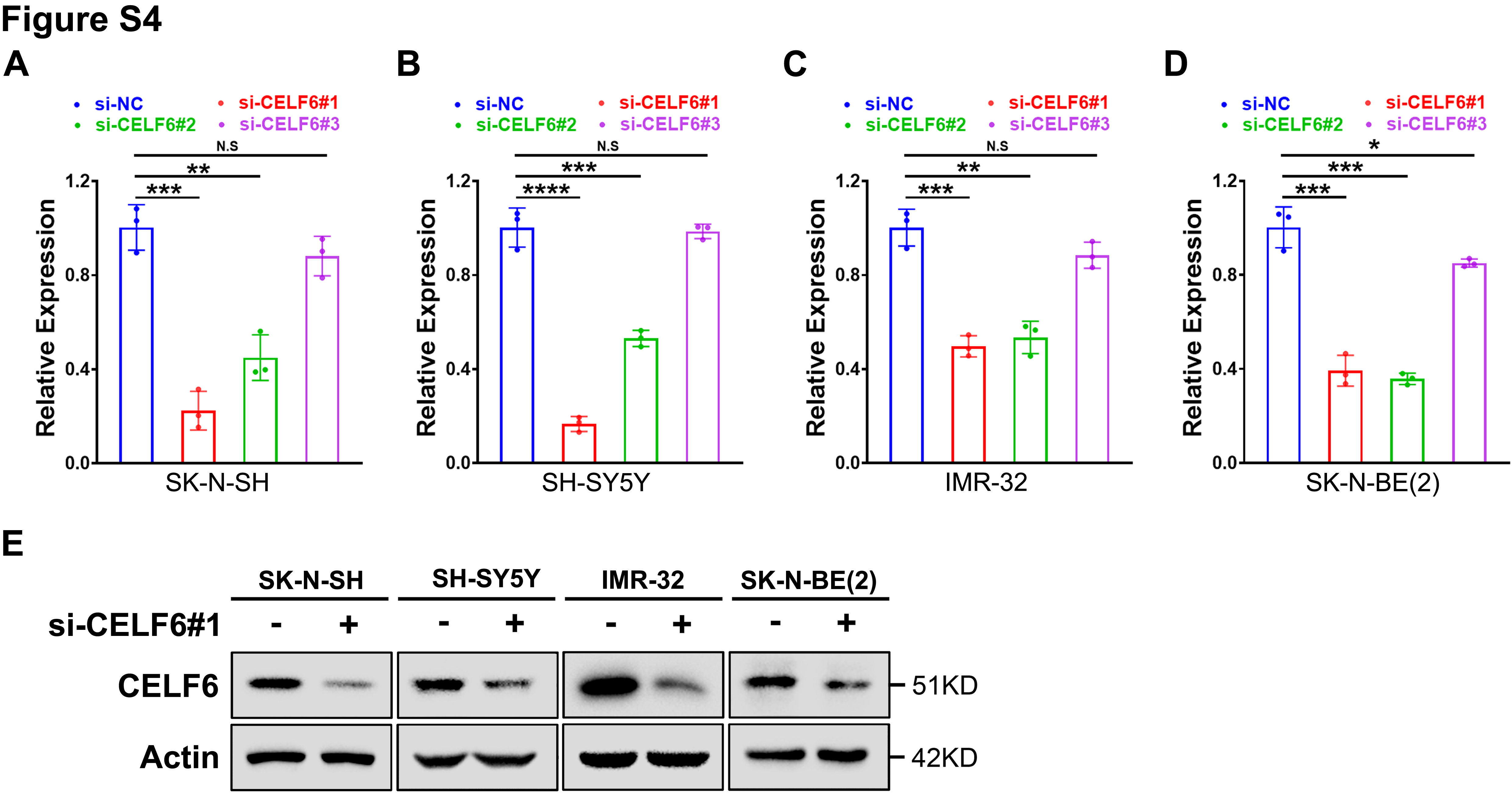


**Figure S4. Gene inhibition efficiency of si-CELF6 in neuroblastoma cells.**

(A-D) Real-time fluorescence quantitative PCR to detect the inhibitory efficiency of si-CELF6 in SH-SY5Y cell line.

(B) Western blotting to detect the inhibitory efficiency of SH-SY5Y in SK-N-SH cell line.

**Supplementary Tables**

**Supplementary Table 1. Primers for gene knockdown (siRNA)**

**Supplementary Table 2. Primers for DNA amplification**

**Supplementary Table 3. Primers for Q-PCR.**

**Supplementary Table 4. Chemicals and antibodies used in this study.**

**Supplementary Table 5. Functional enrichment analysis**

| **Supplementary Table 1. Primers for gene knockdown (siRNA)** | |
| --- | --- |
| **Primer Name** | **Sequence** |
| si-CELF6#1 | GGAGUUUGGUGAUGCGGAACU |
|  | UUCCGCAUCACCAAACUCCUG |
| si-CELF6#2 | CCUUCUGUCUCUUAGUCUAAG |
|  | UAGACUAAGAGACAGAAGGUA |
| si-CELF6#3 | CCGACACGCUCUACAAUAACG |
|  | UUAUUGUAGAGCGUGUCGGAG |
|  |  |
| si-MDM2#1 | GGUUAAUGGUACUAGACAACA |
|  | UUGUCUAGUACCAUUAACCAA |
| si-MDM2#2 | GGAACUUGGUAGUAGUCAAUC |
|  | UUGACUACUACCAAGUUCCUG |
| si-MDM2#3 | GCUUCACAAUCACAAGAAAGU |
|  | UUUCUUGUGAUUGUGAAGCUU |

| **Supplementary Table 2. Primers for DNA amplification** | | | | | | |
| --- | --- | --- | --- | --- | --- | --- |
| **Primer Name** | | **Sequence** | | | | |
| H-CELF6-F | | TAAGGGATCCGAATTC atggccgcggcgccgggagggtcag | | | | |
| H-CELF6-R | | GATGGTGGTGCTCGAG tcagtaaggccggttggcatccttgggc | | | | |
| H-MDM2-F | | TAAGGGATCCGAATTC atgtgcaataccaacatgtctgtac | | | | |
| H-MDM2-R | | GATGGTGGTGCTCGAG ctaggggaaataagttagcacaatcatt | | | | |
| **Supplementary Table 3. Primers for Q-PCR.** | | | | | | |
| **Primer Name** | | | **Sequence** | | | |
| CELF6 | | | CACGCTCTACAATAACGGGC | | | |
|  | | | TGGCTCGATCCACAAAGACT | | | |
| MDM2 | | | ACTGTGCCTGGCCTGATTAT | | | |
|  | | | TGGTCTTCTCAGATGCCTCC | | | |
| 18S | | | GTAACCCGTTGAACCCCATT | | | |
|  | | | CCATCCAATCGGTAGTAGCG | | | |
| **Supplementary Table 4. Chemicals and antibodies used in this study.** | | | | | | |
| **List** | **Antibodies/chemical** | | | **Dilution** | **Vendors** | **Catalog no.** |
| Chemical | Cycloheximide | | | 50 μg/ml | Sigma | 239764 |
|  | MG-132 | | | 10 μM | Sigma | 474790 |
|  | Bortezomib | | | 10 μM | MCE | HY-10227 |
|  | Guanidine hydrochloride | | | 6 mol/L | Sigma | 1302134 |
|  | CD532 | | | 50nM | MCE | HY-112273 |
| Antibody | β-Actin | | | 1:1000 | Transgen | HC201-01 |
|  | Flag | | | 1:2000 | Transgen | HT201-01 |
|  | HA | | | 1:5000 | Transgen | HT301-01 |
|  | p53 | | | 1:1000 | Proteintech | 10442-1-AP |
|  | CELF6 | | | 1:1000 | Abcam | ab173282 |
|  | MDM2 | | | 1:1000 | Abcam | ab259265 |

| **Supplementary Table 5. Functional enrichment analysis** | | | | | |
| --- | --- | --- | --- | --- | --- |
| **Category** | **Term** | **Count** | **%** | ***P*** | ***P* _FDR_** |
| BP | GO:0007411~axon guidance | 6 | 8.33 | 0.00 | 0.19 |
| BP | GO:0007420~brain development | 6 | 8.33 | 0.00 | 0.19 |
| BP | GO:0048791~calcium ion-regulated exocytosis of neurotransmitter | 3 | 4.17 | 0.00 | 0.24 |
| BP | GO:0017158~regulation of calcium ion-dependent exocytosis | 3 | 4.17 | 0.00 | 0.30 |
| BP | GO:0007626~locomotory behavior | 4 | 5.56 | 0.00 | 0.30 |
| BP | GO:0071277~cellular response to calcium ion | 4 | 5.56 | 0.00 | 0.30 |
| BP | GO:0014059~regulation of dopamine secretion | 3 | 4.17 | 0.01 | 0.36 |
| BP | GO:0098609~cell-cell adhesion | 5 | 6.94 | 0.01 | 0.36 |
| BP | GO:0001975~response to amphetamine | 3 | 4.17 | 0.01 | 0.36 |
| BP | GO:0007155~cell adhesion | 7 | 9.72 | 0.01 | 0.41 |
| BP | GO:0006564~L-serine biosynthetic process | 2 | 2.78 | 0.02 | 0.76 |
| BP | GO:0042297~vocal learning | 2 | 2.78 | 0.02 | 0.76 |
| BP | GO:0007416~synapse assembly | 3 | 4.17 | 0.02 | 0.76 |
| BP | GO:0042262~DNA protection | 2 | 2.78 | 0.02 | 0.76 |
| BP | GO:0007157~heterophilic cell-cell adhesion via plasma membrane cell adhesion molecules | 3 | 4.17 | 0.03 | 0.76 |
| BP | GO:0007156~homophilic cell adhesion via plasma membrane adhesion molecules | 4 | 5.56 | 0.03 | 0.76 |
| BP | GO:0032289~central nervous system myelin formation | 2 | 2.78 | 0.03 | 0.76 |
| BP | GO:0019227~neuronal action potential propagation | 2 | 2.78 | 0.03 | 0.89 |
| BP | GO:0032956~regulation of actin cytoskeleton organization | 3 | 4.17 | 0.04 | 1.00 |
| BP | GO:0007268~chemical synaptic transmission | 4 | 5.56 | 0.05 | 1.00 |
| CC | GO:0030424~axon | 11 | 15.28 | 0.00 | 0.00 |
| CC | GO:0042734~presynaptic membrane | 5 | 6.94 | 0.00 | 0.06 |
| CC | GO:0005886~plasma membrane | 31 | 43.06 | 0.00 | 0.06 |
| CC | GO:0005721~pericentric heterochromatin | 3 | 4.17 | 0.00 | 0.19 |
| CC | GO:0030672~synaptic vesicle membrane | 4 | 5.56 | 0.01 | 0.26 |
| CC | GO:0098978~glutamatergic synapse | 6 | 8.33 | 0.01 | 0.29 |
| CC | GO:0008623~CHRAC | 2 | 2.78 | 0.01 | 0.29 |
| CC | GO:0042584~chromaffin granule membrane | 2 | 2.78 | 0.03 | 0.56 |
| CC | GO:0043025~neuronal cell body | 5 | 6.94 | 0.03 | 0.56 |
| CC | GO:0031045~dense core granule | 2 | 2.78 | 0.03 | 0.56 |
| CC | GO:0030667~secretory granule membrane | 3 | 4.17 | 0.04 | 0.62 |
| CC | GO:0098691~dopaminergic synapse | 2 | 2.78 | 0.05 | 0.63 |
| MF | GO:0005544~calcium-dependent phospholipid binding | 5 | 6.94 | 0.00 | 0.01 |
| MF | GO:0030170~pyridoxal phosphate binding | 4 | 5.56 | 0.00 | 0.10 |
| MF | GO:0005509~calcium ion binding | 9 | 12.50 | 0.00 | 0.27 |
| MF | GO:0098632~cell-cell adhesion mediator activity | 3 | 4.17 | 0.01 | 0.45 |
| MF | GO:0030276~clathrin binding | 3 | 4.17 | 0.01 | 0.45 |
| MF | GO:0019905~syntaxin binding | 3 | 4.17 | 0.02 | 0.52 |
| MF | GO:0000149~SNARE binding | 3 | 4.17 | 0.02 | 0.61 |
| MF | GO:0001786~phosphatidylserine binding | 3 | 4.17 | 0.03 | 0.61 |
| MF | GO:0017166~vinculin binding | 2 | 2.78 | 0.04 | 0.88 |
| KEGG | hsa04514:Cell adhesion molecules | 7 | 9.72 | 0.00 | 0.00 |
| KEGG | hsa00260:Glycine, serine and threonine metabolism | 4 | 5.56 | 0.00 | 0.01 |
| KEGG | hsa00270:Cysteine and methionine metabolism | 3 | 4.17 | 0.01 | 0.37 |
| KEGG | hsa01230:Biosynthesis of amino acids | 3 | 4.17 | 0.03 | 0.55 |
| REACTOME | R-HSA-373760~L1CAM interactions | 4 | 5.56 | 0.01 | 1.00 |
| REACTOME | R-HSA-71291~Metabolism of amino acids and derivatives | 6 | 8.33 | 0.02 | 1.00 |
| REACTOME | R-HSA-209905~Catecholamine biosynthesis | 2 | 2.78 | 0.02 | 1.00 |
| REACTOME | R-HSA-447043~Neurofascin interactions | 2 | 2.78 | 0.03 | 1.00 |
| REACTOME | R-HSA-977347~Serine biosynthesis | 2 | 2.78 | 0.04 | 1.00 |
| REACTOME | R-HSA-1430728~Metabolism | 15 | 20.83 | 0.04 | 1.00 |
| REACTOME | R-HSA-421270~Cell-cell junction organization | 3 | 4.17 | 0.05 | 1.00 |
