# Supplementary material for: MDM2 promotes CELF6 ubiquitination-dependent degradation to promote neuroblastoma cell proliferation
Source: Cell Death Dis. 2025 Oct 21;16(1):736. doi: 10.1038/s41419-025-08048-3 (PMC12540984; doi:10.1038/s41419-025-08048-3)

Figure 2

Figure 2H

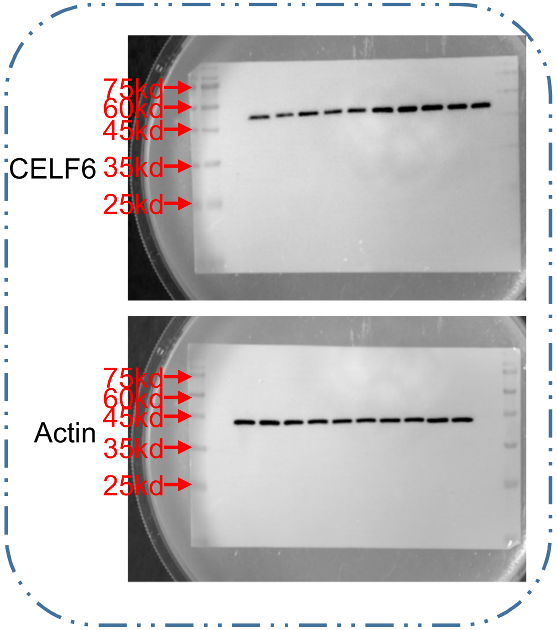

Figure 3

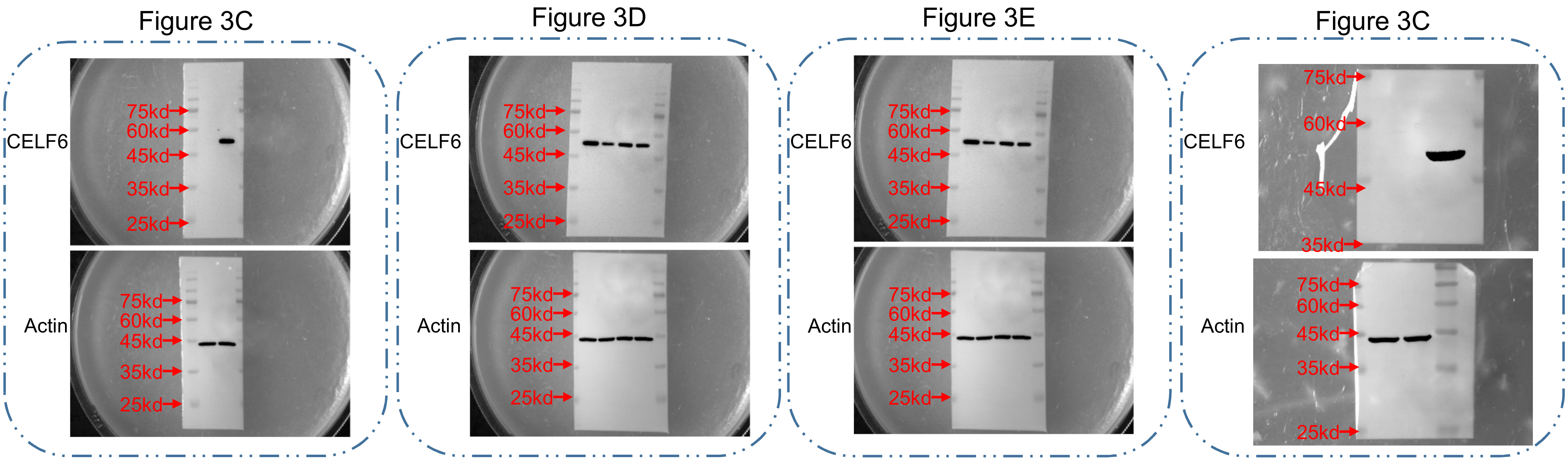

Figure 3C
Figure 3D
Figure 3E
Figure 3C
CELF6
75kd
60kd
45kd
35kd
25kd
Actin

Figure 4

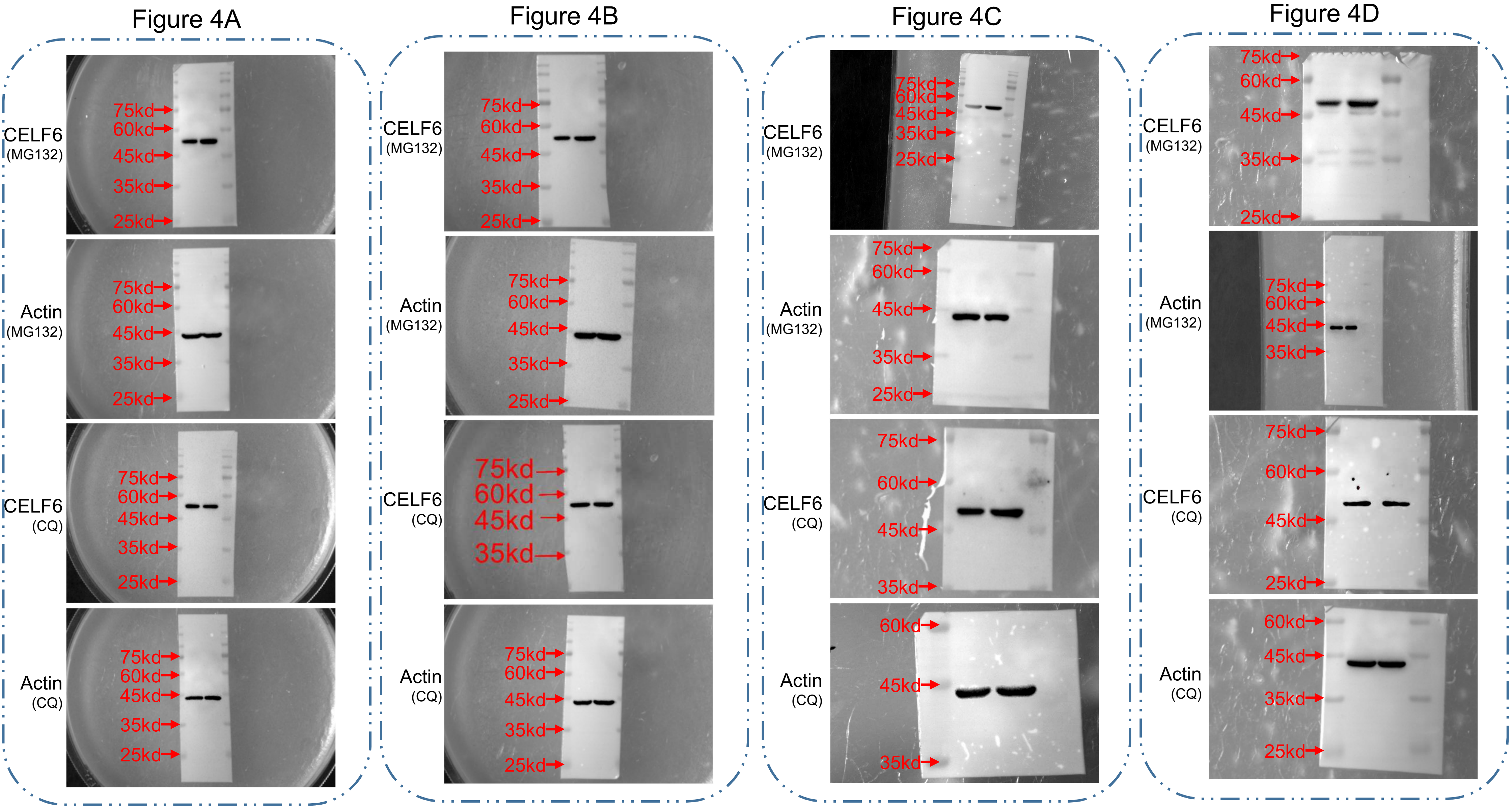
Figure 4A
CELF6 (MG132)
75kd
60kd
45kd
35kd
25kd
Actin (MG132)
75kd
60kd
45kd
35kd
25kd
CELF6 (CQ)
75kd
60kd
45kd
35kd
25kd
Actin (CQ)
75kd
60kd
45kd
35kd
25kd
Figure 4B
CELF6 (MG132)
75kd
60kd
45kd
35kd
25kd
Actin (MG132)
75kd
60kd
45kd
35kd
25kd
CELF6 (CQ)
75kd
60kd
45kd
35kd
Actin (CQ)
75kd
60kd
45kd
35kd
25kd
Figure 4C
CELF6 (MG132)
75kd
60kd
45kd
35kd
25kd
Actin (MG132)
75kd
60kd
45kd
35kd
25kd
CELF6 (CQ)
75kd
60kd
45kd
35kd
Actin (CQ)
60kd
45kd
35kd
Figure 4D
CELF6 (MG132)
75kd
60kd
45kd
35kd
25kd
Actin (MG132)
75kd
60kd
45kd
35kd
CELF6 (CQ)
75kd
60kd
45kd
25kd
Actin (CQ)
60kd
45kd
35kd
25kd

Figure 4

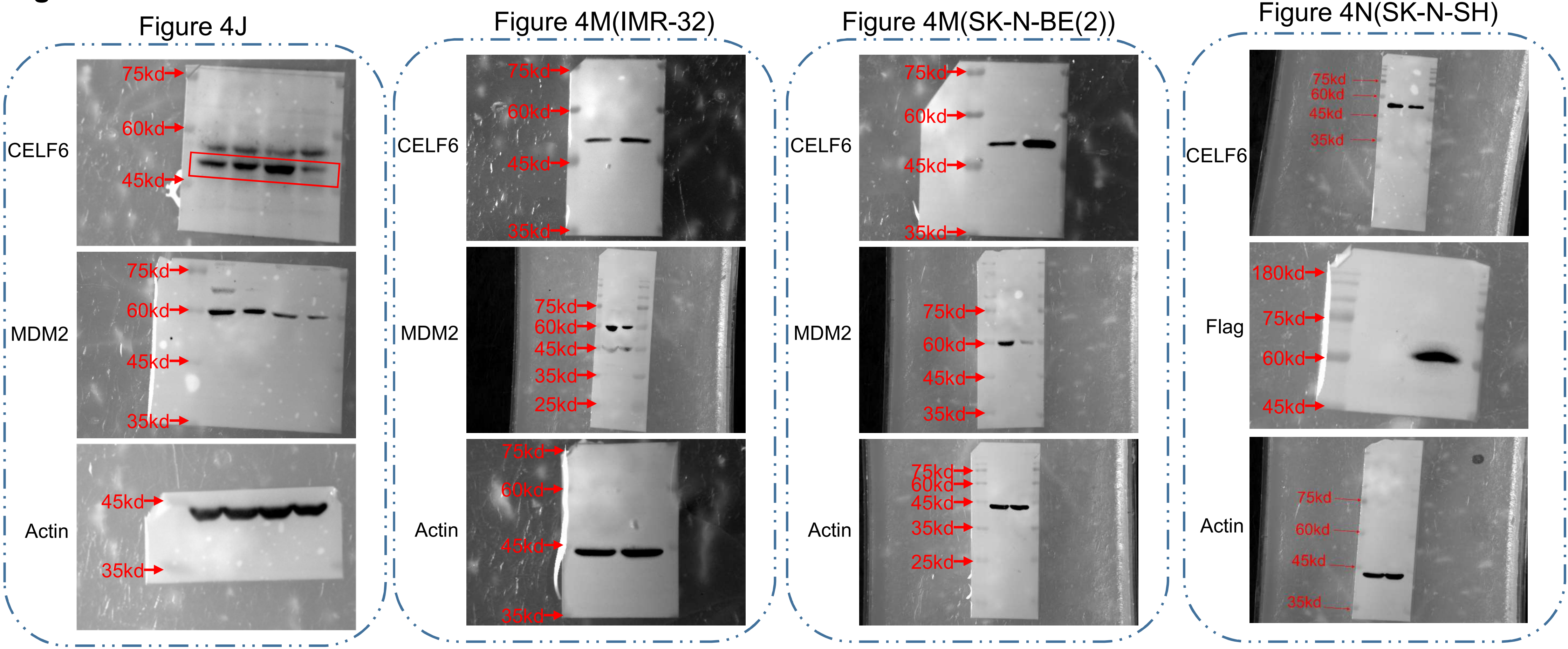

Figure 4J
CELF6
75kd
60kd
45kd
MDM2
75kd
60kd
45kd
35kd
Actin
45kd
35kd
Figure 4M(IMR-32)
CELF6
75kd
60kd
45kd
35kd
MDM2
75kd
60kd
45kd
35kd
25kd
Actin
75kd
60kd
45kd
35kd
Figure 4M(SK-N-BE(2))
CELF6
75kd
60kd
45kd
35kd
MDM2
75kd
60kd
45kd
35kd
Actin
75kd
60kd
45kd
35kd
25kd
Figure 4N(SK-N-SH)
CELF6
75kd
60kd
45kd
35kd
Flag
180kd
75kd
60kd
45kd
Actin
75kd
60kd
45kd
35kd

Figure 4

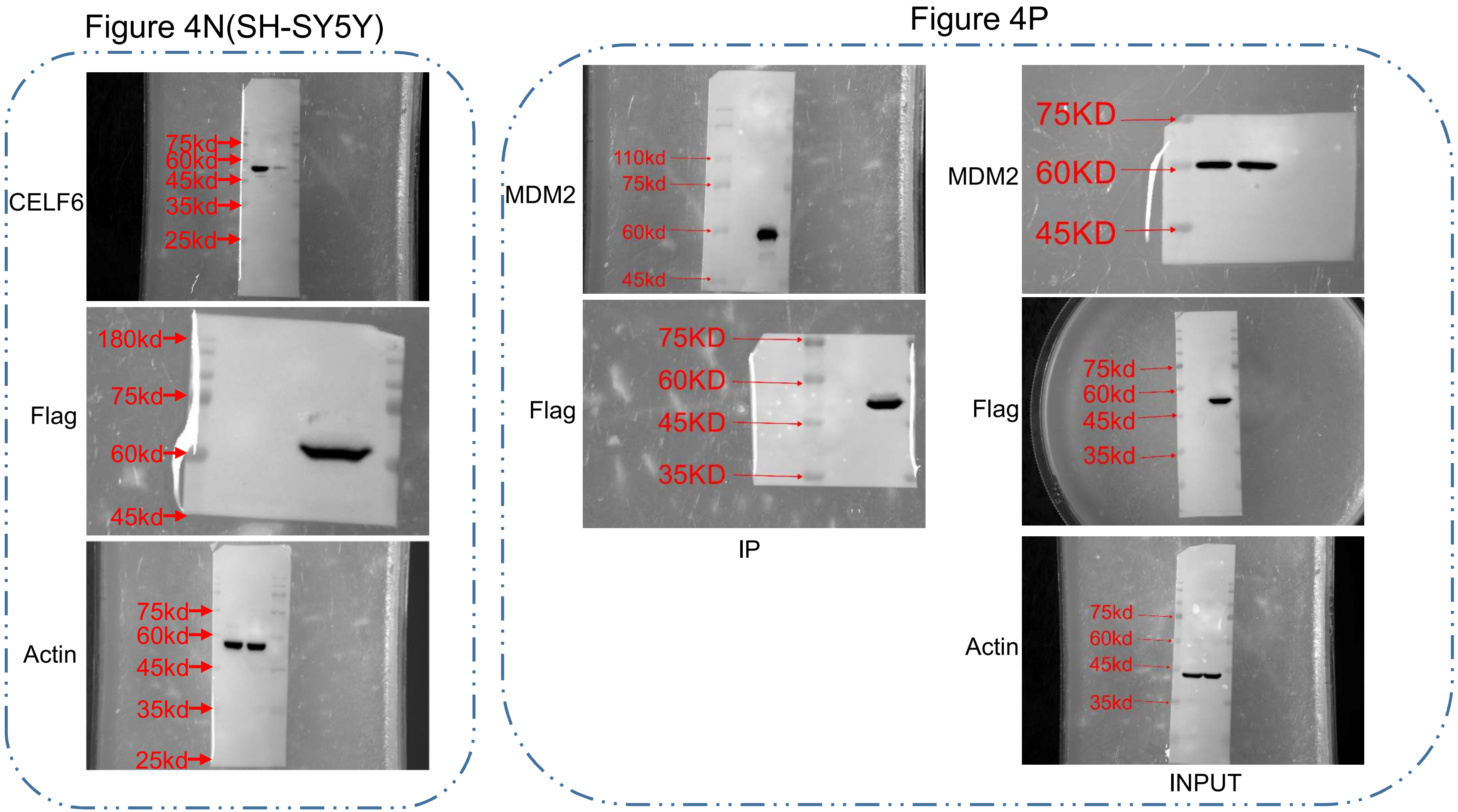

Figure 4N(SH-SY5Y)
CELF6
75kd
60kd
45kd
35kd
25kd
Flag
180kd
75kd
60kd
45kd
Actin
75kd
60kd
45kd
35kd
25kd
Figure 4P
MDM2
110kd
75kd
60kd
45kd
Flag
75KD
60KD
45KD
35KD
IP
MDM2
75KD
60KD
45KD
Flag
75kd
60kd
45kd
35kd
Actin
75kd
60kd
45kd
35kd
INPUT

Figure 4

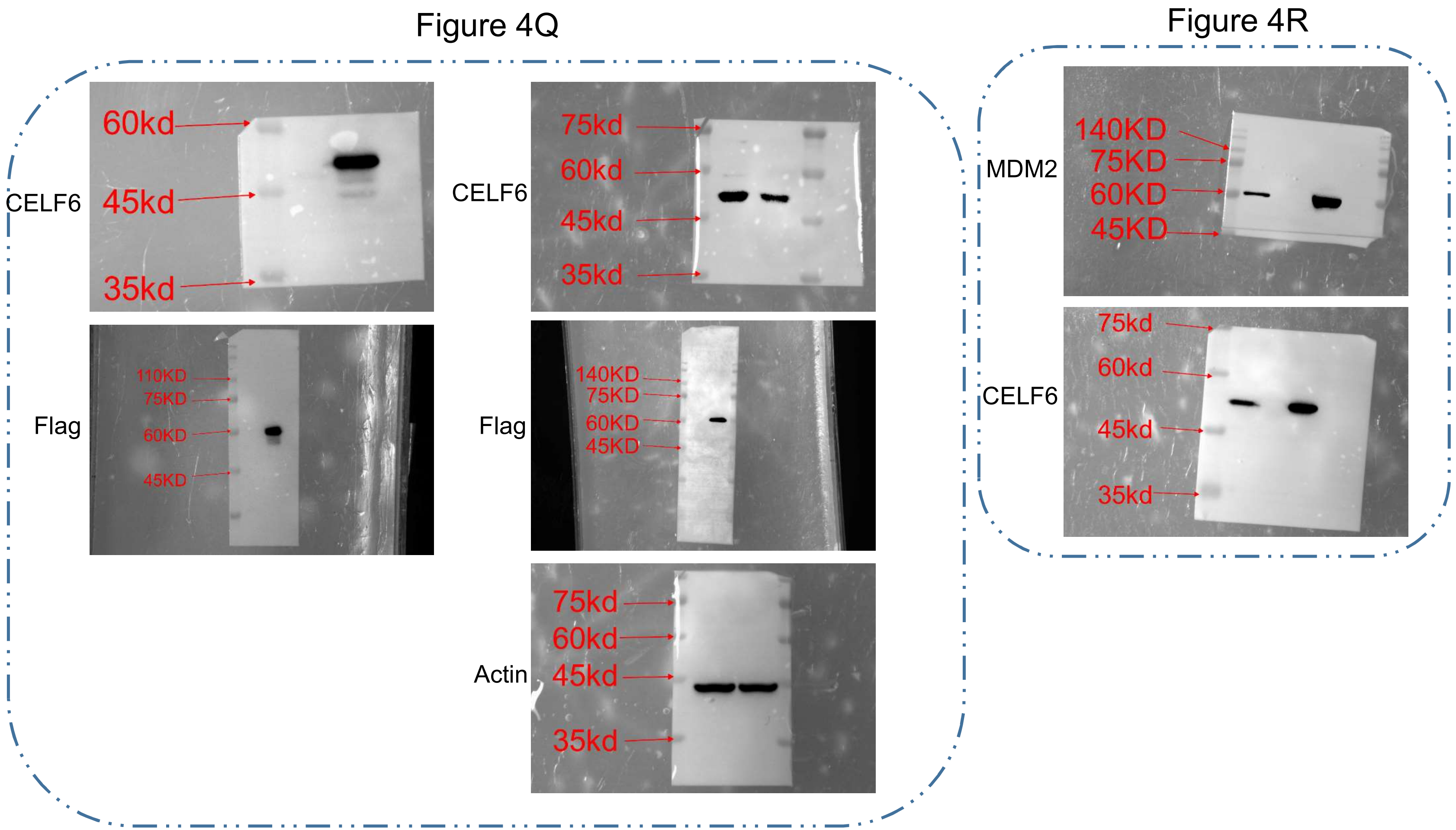
Figure 4Q
CELF6
60kd
45kd
35kd
CELF6
75kd
60kd
45kd
35kd
Flag
110KD
75KD
60KD
45KD
Flag
140KD
75KD
60KD
45KD
Actin
75kd
60kd
45kd
35kd
Figure 4R
MDM2
140KD
75KD
60KD
45KD
CELF6
75kd
60kd
45kd
35kd

Figure 5

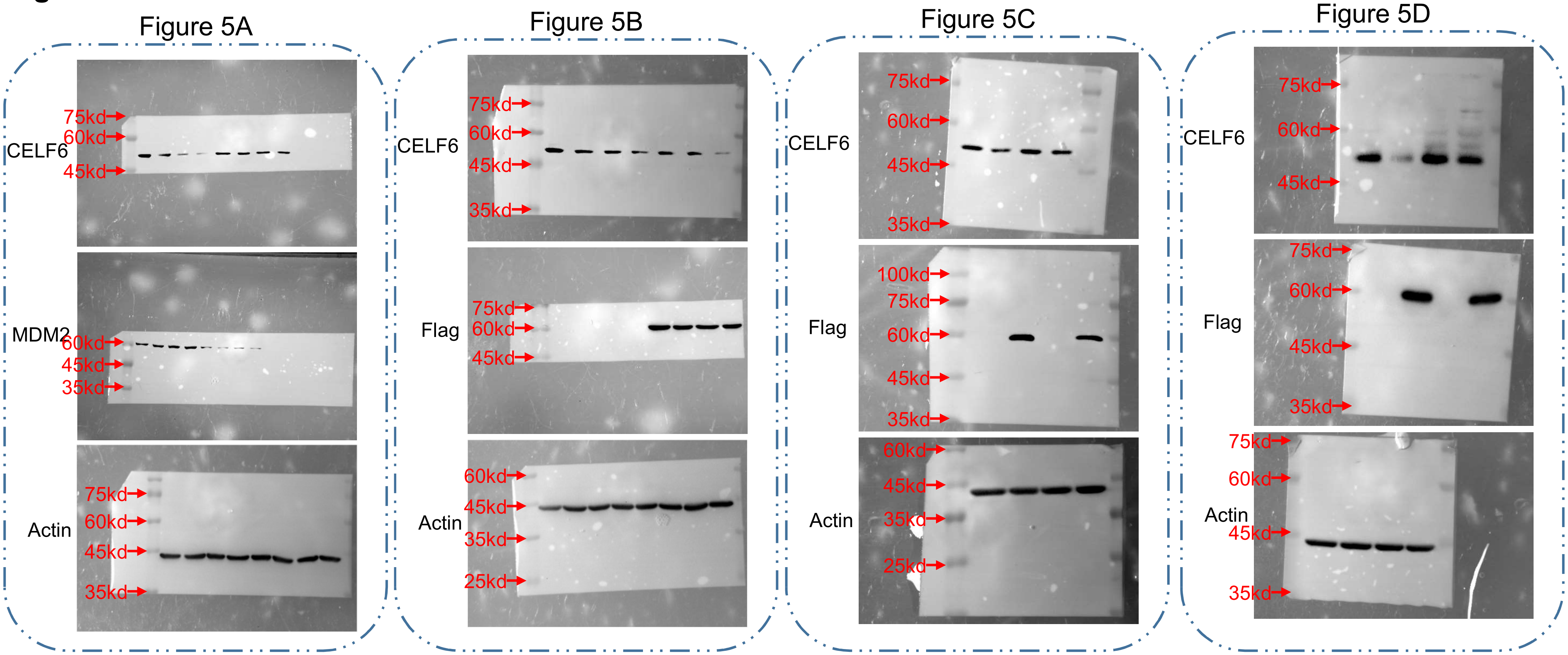

Figure 5A
CELF6
75kd
60kd
45kd
MDM2
60kd
45kd
35kd
Actin
75kd
60kd
45kd
35kd
Figure 5B
CELF6
75kd
60kd
45kd
35kd
Flag
75kd
60kd
45kd
Actin
60kd
45kd
35kd
25kd
Figure 5C
CELF6
75kd
60kd
45kd
35kd
Flag
100kd
75kd
60kd
45kd
35kd
Actin
60kd
45kd
35kd
25kd
Figure 5D
CELF6
75kd
60kd
45kd
Flag
75kd
60kd
45kd
35kd
Actin
75kd
60kd
45kd
35kd

Figure 5

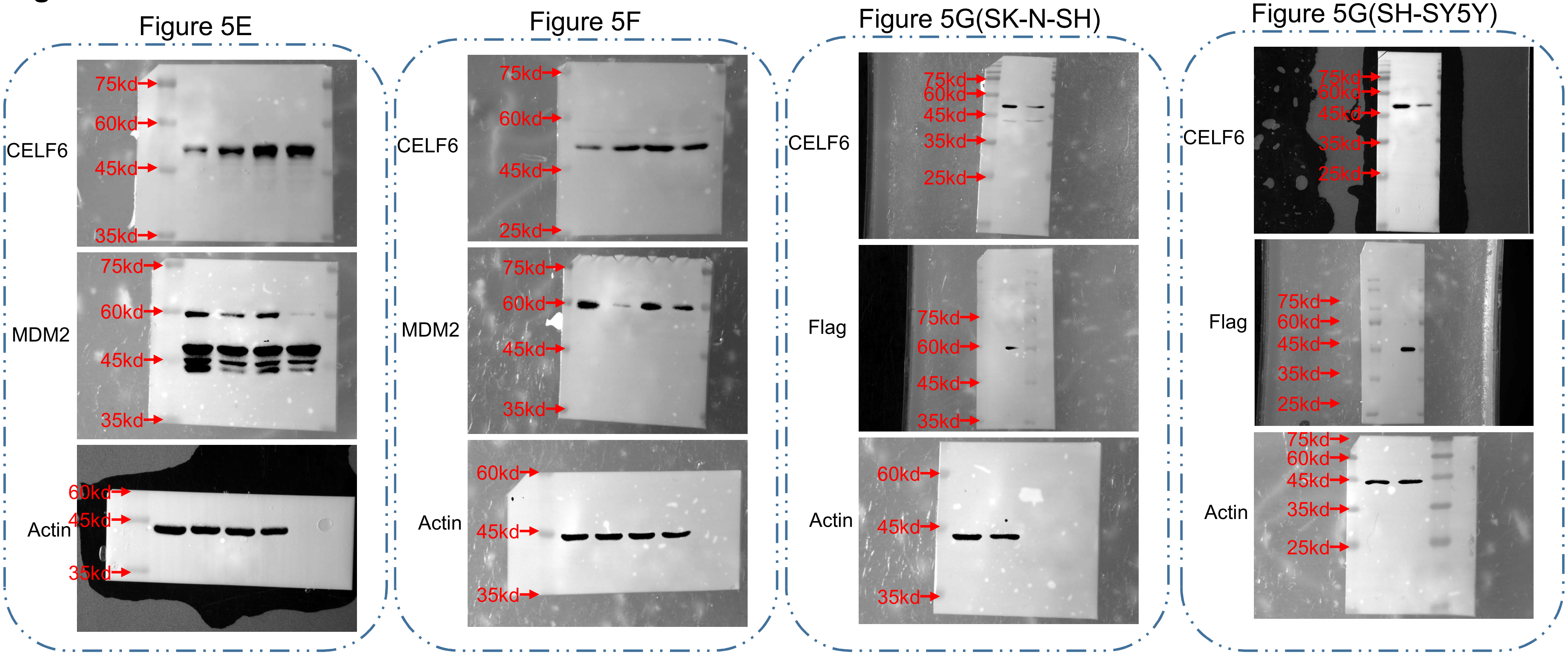
Figure 5E
CELF6
75kd
60kd
45kd
35kd
MDM2
75kd
60kd
45kd
35kd
Actin
60kd
45kd
35kd
Figure 5F
CELF6
75kd
60kd
45kd
25kd
MDM2
75kd
60kd
45kd
35kd
Actin
60kd
45kd
35kd
Figure 5G(SK-N-SH)
CELF6
75kd
60kd
45kd
35kd
25kd
Flag
75kd
60kd
45kd
35kd
Actin
60kd
45kd
35kd
Figure 5G(SH-SY5Y)
CELF6
75kd
60kd
45kd
35kd
25kd
Flag
75kd
60kd
45kd
35kd
25kd
Actin
75kd
60kd
45kd
35kd
25kd

Figure 5

Figure 5H(IMR-32)

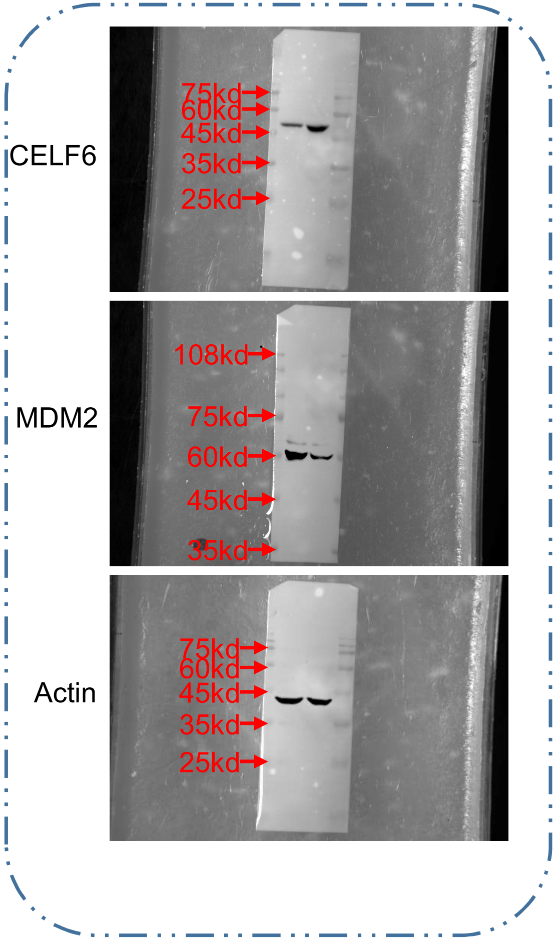

Figure 5H(SK-N-BE(2))

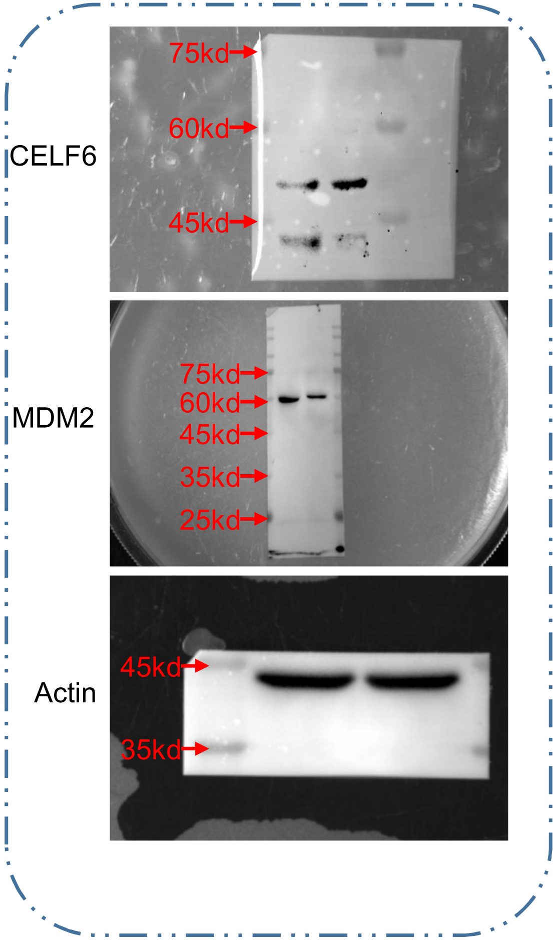

Figure 5I

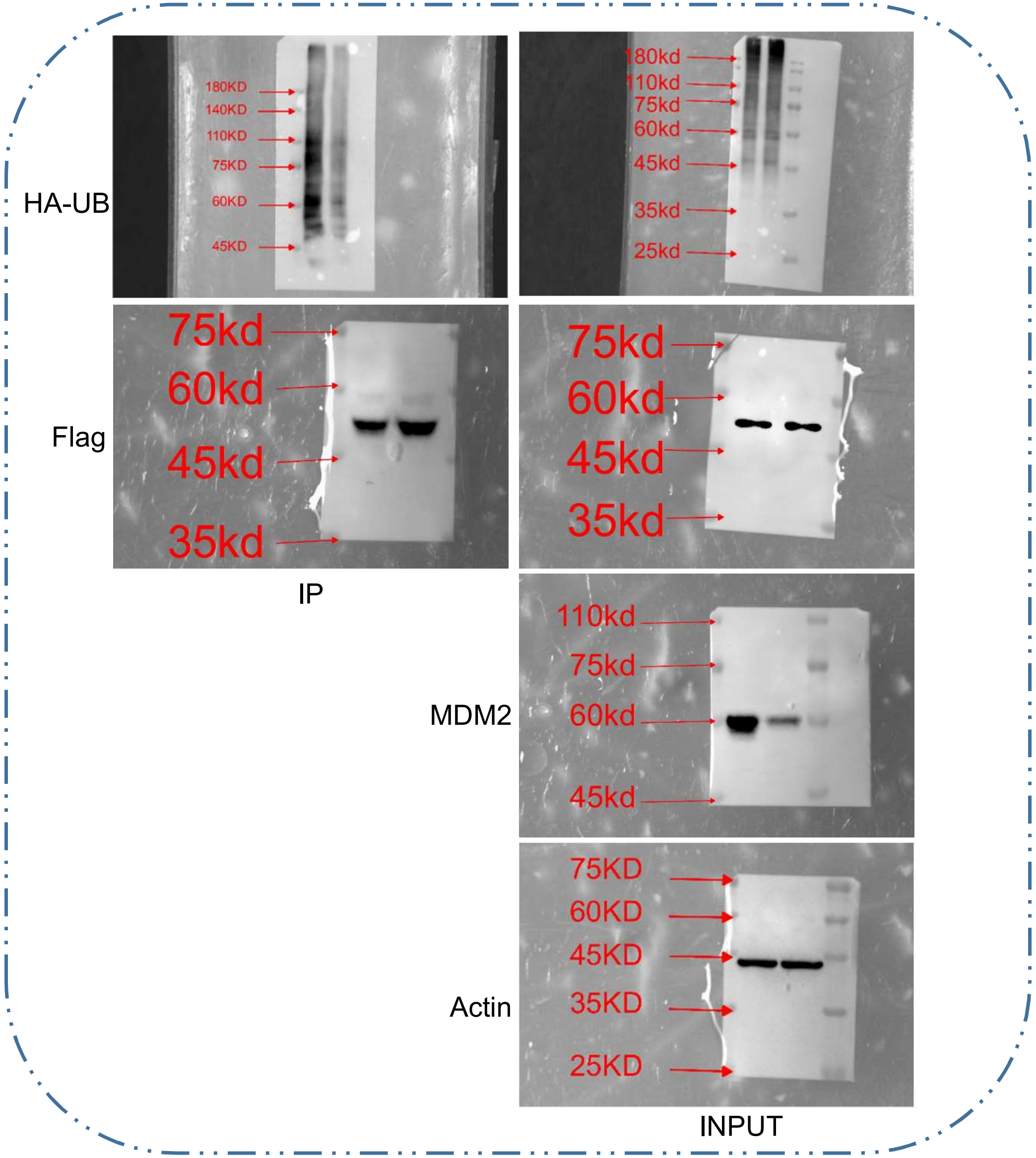

Figure 5

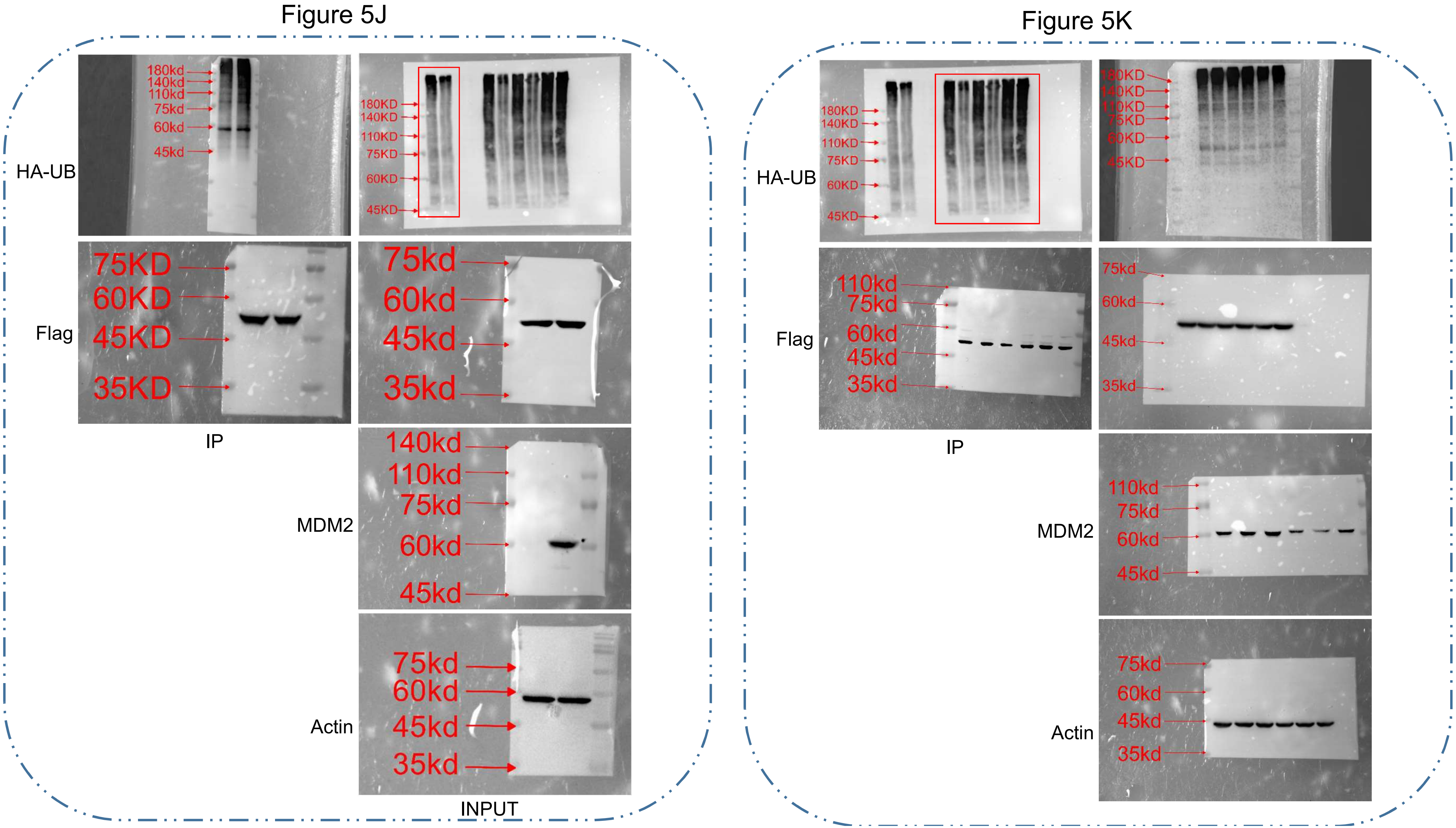
Figure 5J
HA-UB
180kd
140kd
110kd
75kd
60kd
45kd
180KD
140KD
110KD
75KD
60KD
45KD
Flag
75KD
60KD
45KD
35KD
75kd
60kd
45kd
35kd
IP
MDM2
140kd
110kd
75kd
60kd
45kd
Actin
75kd
60kd
45kd
35kd
INPUT
Figure 5K
HA-UB
180KD
140KD
110KD
75KD
60KD
45KD
Flag
110kd
75kd
60kd
45kd
35kd
75kd
60kd
45kd
35kd
IP
MDM2
110kd
75kd
60kd
45kd
Actin
75kd
60kd
45kd
35kd

Figure S2

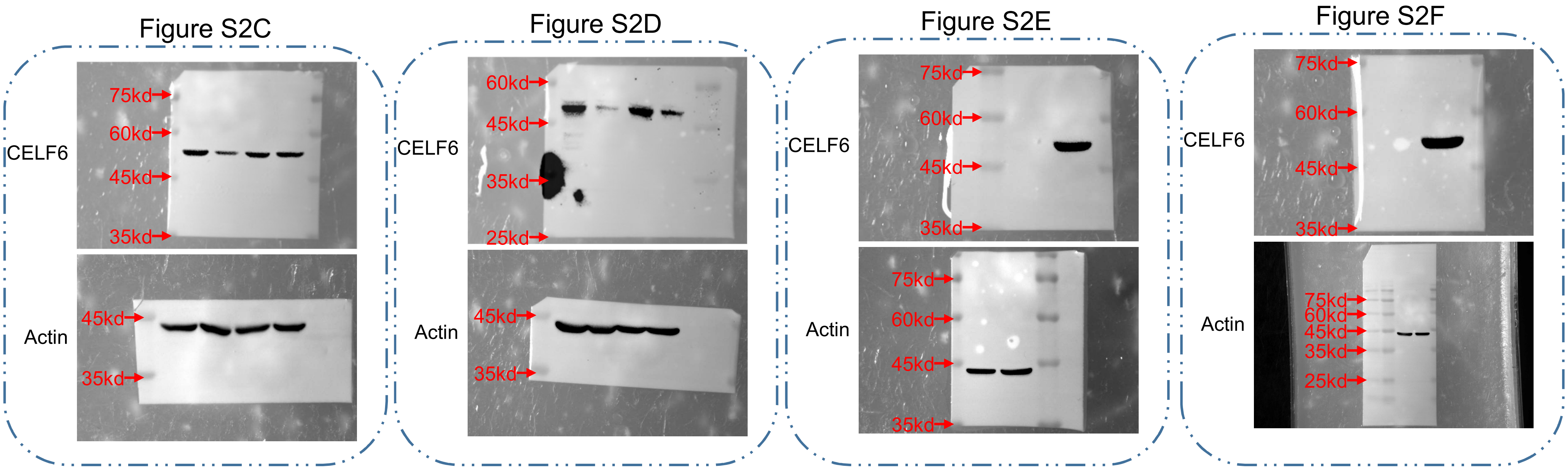
Figure S2C
CELF6
75kd
60kd
45kd
35kd
Actin
45kd
35kd
Figure S2D
CELF6
60kd
45kd
35kd
25kd
Actin
45kd
35kd
Figure S2E
CELF6
75kd
60kd
45kd
35kd
Actin
75kd
60kd
45kd
35kd
Figure S2F
CELF6
75kd
60kd
45kd
35kd
Actin
75kd
60kd
45kd
35kd
25kd

Figure S3

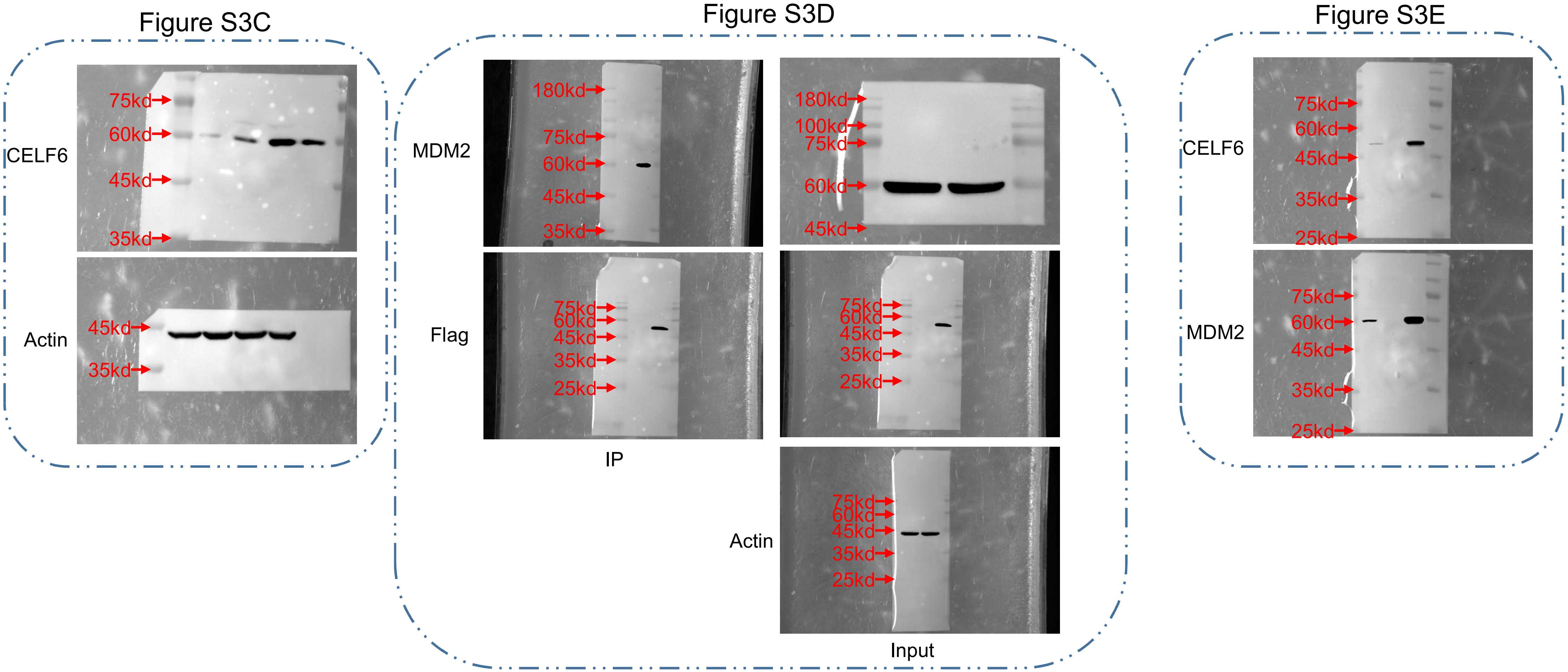
Figure S3C
CELF6
75kd
60kd
45kd
35kd
Actin
45kd
35kd
Figure S3D
MDM2
180kd
75kd
60kd
45kd
35kd
180kd
100kd
75kd
60kd
45kd
Flag
75kd
60kd
45kd
35kd
25kd
IP
Actin
75kd
60kd
45kd
35kd
25kd
Input
Figure S3E
CELF6
75kd
60kd
45kd
35kd
25kd
MDM2
75kd
60kd
45kd
35kd
25kd

Figure S4

Figure S4E

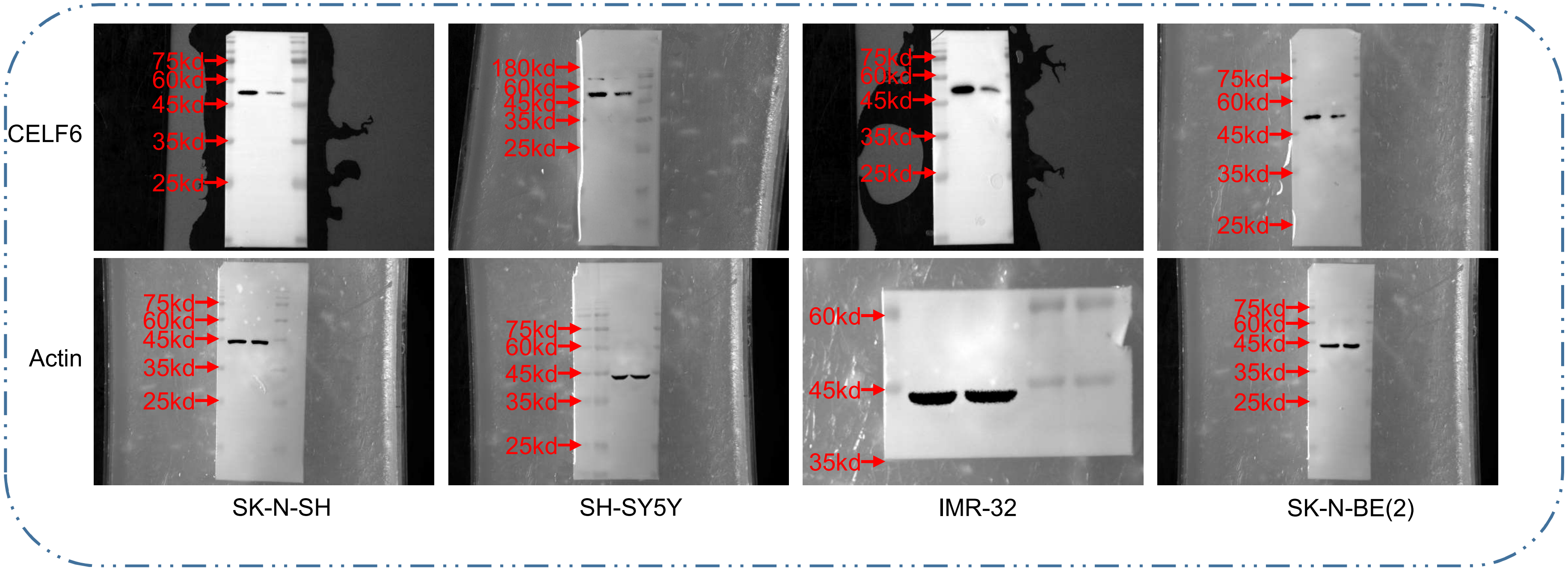

Supplement: Supplementary file 2 — Original Data [file 41419_2025_8048_MOESM2_ESM.pdf]
